# Two novel Patescibacteria: *Phycocordibacter aenigmaticus* gen. nov. sp. nov. and *Minusculum obligatum* gen. nov. sp. nov., both associated with microalgae optimized for carbon dioxide sequestration from flue gas

**DOI:** 10.1128/mbio.01231-25

**Published:** 2025-06-12

**Authors:** Lauren Jonas, Yi-Ying Lee, Tsvetan Bachvaroff, Russell T. Hill, Yantao Li

**Affiliations:** 1 Institute of Marine and Environmental Technology 145781 , Baltimore, Maryland, USA; 2 University of Maryland Center for Environmental Science, Baltimore, Maryland, USA; Oregon State University, Corvallis, Oregon, USA

**Keywords:** Patescibacteria, microalgae, *Minusculum*, *Phycocordibacter*, metabolic limitation, reduced genome

## Abstract

The functional roles of bacterial symbionts associated with microalgae remain understudied despite the importance of microalgae in biotechnology and environmental microbiology. 16S rRNA gene sequencing was conducted to analyze bacterial communities associated with two microalgae optimized for growth with flue gas containing 5%–10% CO_2_. Two dominant bacteria with no taxonomic classification beyond the class level (Paceibacteria) were discovered repeatedly in the most productive algal cultures. Long-read metagenomic sequencing was conducted to yield high-quality metagenomes, from which two novel species were discovered under the Seqcode (seqco.de/r:ywe1blo2), *Phycocordibacter aenigmaticus* gen. nov. sp. nov. and *Minusculum obligatum* gen. nov. sp. nov. The genus *Phycocordibacter* gen. nov. was proposed as the nomenclatural type of the family Phycocordibacteraceae fam. nov. and the order Phycocordibacterales ord. nov. Both bacteria possessed features typical of Patescibacteria such as reduced genomes (<800 kbp), lack of complete glycolysis and tricarboxylic acid (TCA) cycle pathways, and inability to synthesize amino acids. Instead, they rely on the reductive pentose phosphate pathway (Calvin cycle) for essential biosynthesis and redox balance. *P. aenigmaticus* may also rely on elemental sulfur oxidation (*sdo*), partial nitrite reduction (*nirK*), and sulfur-related amino acid metabolism (SAMe → SAH). Both bacteria were found in high relative abundance in cultures of *Tetradesmus obliquus* HTB1 (freshwater) and *Nannochloropsis oceanica* IMET1 (marine), suggesting a tight association with microalgae in various environments. The absence of full metabolic pathways for energy production suggests extreme metabolic limitations and obligate symbiosis, most likely with other bacteria associated with the microalgae.

**IMPORTANCE:**

To our knowledge, this is the first report of Patescibacteria as dominant bacteria associated with microalgae or within a biologically mediated carbon capture system. Two novel Patescibacteria were found in two ecologically distinct microalgal cultures (one freshwater strain and one marine) regardless of whether the cultures were bubbled with air, 5% CO_2_, or 10% CO_2_. This unexpected and unprecedented dominance led to long-read sequencing and the assembly of high-quality metagenomes for both Patescibacteria, as well as five other bacteria in the system. The discovery of two novel species belonging to two novel genera, one novel family, and one novel order has enabled us to fill in gaps of a major, uncharacterized branch within the bacterial tree of life. Additionally, the extreme gene loss found in both Patescibacteria, *Phycocordibacter aenigmaticus* and *Minusculum obligatum*, contributes knowledge to a rapidly advancing body of research on the scavenging metabolic nature of this enigmatic and largely unclassified phylum.

## INTRODUCTION

Microalgae are significant players in the capture and transformation of greenhouse gases, particularly carbon dioxide (CO_2_). Moreover, the bacterial symbionts of microalgae are crucial for culture growth and resilience, leading to more beneficial carbon capture technologies. The significance of bacteria within microalgal systems is a significantly undervalued factor in microalgal research despite growing evidence of various benefits that bacterial cells can supply to their hosts (1).

The phycosphere is a hotspot for symbiosis due to a high concentration of fixed organic carbon compounds that stimulate prokaryotic growth. Bacteria have been shown to provide phytohormones and B vitamins in exchange for fixed carbon (2), and both organisms have been found to alter their metabolism to benefit one another. For example, *Sulfitobacter* sp. was shown to provide the phytohormone indole-3-acetic acid (IAA) in exchange for algal-excreted organosulfur molecules as well as tryptophan, which then served as a substrate for more IAA production (3). The positive feedback of both organisms has allowed for microalgal-bacterial consortia to be leveraged for various bioremediation strategies such as improved wastewater treatment (4).

Recent efforts in long-read metagenomics sequencing have allowed for the reconstruction of prokaryotic genomes, many of which make up what is known as “microbial dark matter.” It has been estimated that >99% of microbes have yet to be formally named, leading to taxonomic voids dispersed across the prokaryotic tree of life (5). The description of novel taxonomic groups is significant not only to fill in these gaps but can also unveil unusual biological traits. One example is the hyper-reduced genome size and metabolic incapacity of many members of the vastly uncharacterized phylum, Patescibacteria.

High-quality deep metagenomic sequencing of Patescibacteria has been constrained because these bacteria are generally present as minor players in complex bacterial communities. Patescibacteria are largely uncultivated, although some have been obtained in co-culture and are obligate symbionts that attach to bacterial or archaeal host cell surfaces (6–9). There are additional examples of members of Candidate Phylum Radiation (CPR)—a group that often includes Patescibacteria (10)—living as parasitic epibionts of other bacteria (11, 12). From the metagenomic sequence data that are available, some consistent patterns have emerged across members of Patescibacteria, including their cosmopolitan distribution (13, 14), small cell size (15, 16), ultra-reduced genomes (17, 18), and highly limited metabolic capabilities (13, 19). Most members of Patescibacteria are unable to synthesize most (or any) amino acids, and all members lack complete gene pathways for electron transport chains and the TCA cycle, leading to the hypothesis that associations with more complex organisms may be necessary for their survival (18, 20). Limited metabolic function and predicted symbiotic lifestyles in Patescibacteria and lower phylogenetic ranks have been found to span large redox gradients from fully oxic to completely anoxic environments (20). Despite their gene absence, studies have reported Patescibacteria to be contributing members in various environments and bioremediation technologies (21, 22).

Here, we gained insights into two novel Patescibacteria, facilitated by their unprecedented high relative abundance in the microbiome of two microalgal species, *Tetradesmus obliquus* HTB1 (freshwater alga) and *Nannochloropsis oceanica* IMET1 (marine), both optimized to grow under high (5–10%) CO_2_ conditions (simulated flue gas). Because of the dominance of both Patescibacteria in our 500 L bioreactors, we were able to recover high-quality metagenome-assembled genomes (MAGs) with high coverage from long-read sequence data. Here, we describe and characterize *Phycocordibacter aenigmaticus* gen. nov. sp. nov. whose genus serves as the nomenclature type for Phycocordibacteraceae fam. nov. and Phycocordibacterales ord. nov. The second bacterium, *Minusculum obligatum* gen. nov. sp. nov., belongs to a second novel genus and falls within the same family and order. Phylogenetic analysis, lifestyle predictions, and metabolic reconstruction were conducted on *P. aenigmaticus*, *M. obligatum*, and five other MAGs associated with the same microalgae.

## MATERIALS AND METHODS

### Algal culture filtration, DNA extraction, and 16S rRNA gene sequencing and analysis

Methods regarding size fractionation, DNA extraction (Qiagen Ultraclean kit), sequencing (Illumina Miseq), and bioinformatic analysis of the V3/V4 region of the 16S rRNA gene (QIIME2) have been described previously (23, 24).

### Metagenomics library preparation and sequencing

DNA concentrations were checked by absorbance at 260 nm using a UV-visible spectrophotometer (Nanodrop) and fluorometry (Qubit 1× dsDNA Assay Kit + Qubit fluorometer) and adjusted to the ideal starting quantity of 100–200 fmol (~1,000 ng) in 47 µL nuclease-free water. DNA was prepared into sequencing libraries using the Ligation Sequencing Kit V14 (Oxford Nanopore) following the manufacturer’s protocol with the following adjustments: during the last step of both the End-Prep and Adapter Ligation and Clean-up protocols, tubes containing AMPure XP beads and the end-prep reaction were incubated at 37°C for 2 min before eluate was removed. Libraries were made up to 12 µL at 10–20 fmol and loaded onto the GridION sequencing platform (Oxford Nanopore) strictly following the manufacturer’s protocol.

### Assembly of MAGs

Reads were base called on the instrument using Dorado and were visualized for length and quality using NanoPlot and filtered using NanoFilt (25). Adapter sequences were removed with Porechop, and sequences were assembled using Flye (v2.9.3) twice: once using the metagenomics parameter (--meta) and once without (26). The best assemblies were chosen for further analysis based on the number of contigs and the N50 value. Coverage was visualized with the Integrative Genome Viewer by overlaying the sorted.bam file on top of the assembly.fasta output (27, 28). Circular contigs with high coverage were extracted for annotation and checked for completeness using CheckM2 (29). Adapting an existing KBase narrative (30), MaxBin2 (v2.2.4) and CONCOCT (v1.1) were used for binning (31, 32), RASTtk (v1.073) was used for annotating multiple microbial assemblies (33), and GTDB-Tk (v2.4.0) was used for taxonomic classification (34, 35). Bins that were >80% complete with <10% contamination were selected for further analysis. Assemblies were extracted from binned contigs within KBase and were uploaded to the Bacterial and Viral Bioinformatics Resource Center (BV-BRC) (36). Fasta files of circular contigs from the original Flye assembly were also uploaded. The Genome Annotation Service uses the RASTtk tool kit to annotate bacterial genomes using the genome of the closest relative (33).

### Phylogenetic analysis

Phylogenetic analysis using the full-length 16S rRNA gene extracted from genomes was carried out with the Type Strain Genome Server (TYGS) and programs therein (37), which queries against formally named organisms. To include sequences from uncultivated bacteria that have not been formally classified, the full-length 16S rRNA gene was also aligned with that of nearest neighbors using NCBI’s BLASTn program. Multigene-based phylogenetic trees were inferred using 120 single-copy concatenated protein markers using the GTDB-Tk Classify workflow, the Interactive Tree of Life (iTOL) online tool, and Adobe Illustrator (38).

### Novelty determination and nomenclature

MAGs were established as high-quality by following the Minimum Information about a Metagenome-Assembled Genome (MIMAG) criteria and standards set forth by experts in the field (39, 40). Size, L50 values, N50 values, coverage, and GC ratio were determined by Flye assembly. There are no generally accepted standardized cutoff values for determining taxonomic ranks for uncultivated bacteria, but instead, researchers are encouraged to compare metrics across several bioinformatic programs to establish a consensus on novelty (39). The Microbial Genomes Atlas (MiGA v1.2.60) was used to determine genome distance metrics based on amino acid identity (AAI) and average nucleotide identity (ANI) against the TypeMat database (41). TYGS was used to calculate digital DNA-DNA hybridization (dDDH) values. MAGs were taxonomically placed using the GTDB-Tk Classify workflow, which compared our MAGs against a mash database composed of all GTDB representative genomes (34, 35). The program then created a multiple sequence alignment using Prodigal (42) and hidden Markov models (HMM) using the HMMER package (43) to identify the 120 bacterial marker genes used for the phylogenetic inference (44). Finally, the classifying step uses pplacer (45) to find the maximum-likelihood placement of each genome in the GTDB-Tk reference tree. To ensure correct placement, only high-quality MAGs with high coverage were used for this analysis. The novelty determination by the GTDB was then supported with other programs such as MiGA that determine taxonomic novelty with AAI% values compared with the other genomes in the database (TypeMat) (41). MiGA then calculates p-values that are estimated from the empirical distribution observed in all the reference genomes at each taxonomic level within the RefSeq database through NCBI. These metrics, along with relative evolutionary divergence (RED) scores provided by GTDB-Tk, were uploaded to the SeqCode in Validation Path 1 (seqco.de/r:ywe1blo2) (46, 47). Nomenclature was proposed based on MAG features and relation to microalgae.

### Metabolic pathway and lifestyle prediction

Prodigal (v2.6.3) in Normal mode was used to perform protein-coding gene prediction, creating input .faa files. The symbiont classifier, symcla (v0.1.0), was used to predict the lifestyle of each MAG, and bacteria are classified as Free-living, Symbiont:Host-associated, or Symbiont:Intracellular (https://github.com/NeLLi-team/symcla). To analyze the metabolic potential of each MAG, METABOLIC-G (v4.0) (48) was used by applying HMMs to various databases (Kyoto Encyclopedia of Genes and Genomes [KEGG], Uniref, Kofam Pfam, and METABOLIC Custom) and using an -m-cutoff of 0.75, resulting in KEGG modules being labeled as “present” if 75% or more of the KEGG module steps are present. Heatmaps were created in R with pheatmap.

## RESULTS

### Bacterial communities associated with two microalgae reveal the dominance of two Patescibacteria

**Fig 1:**
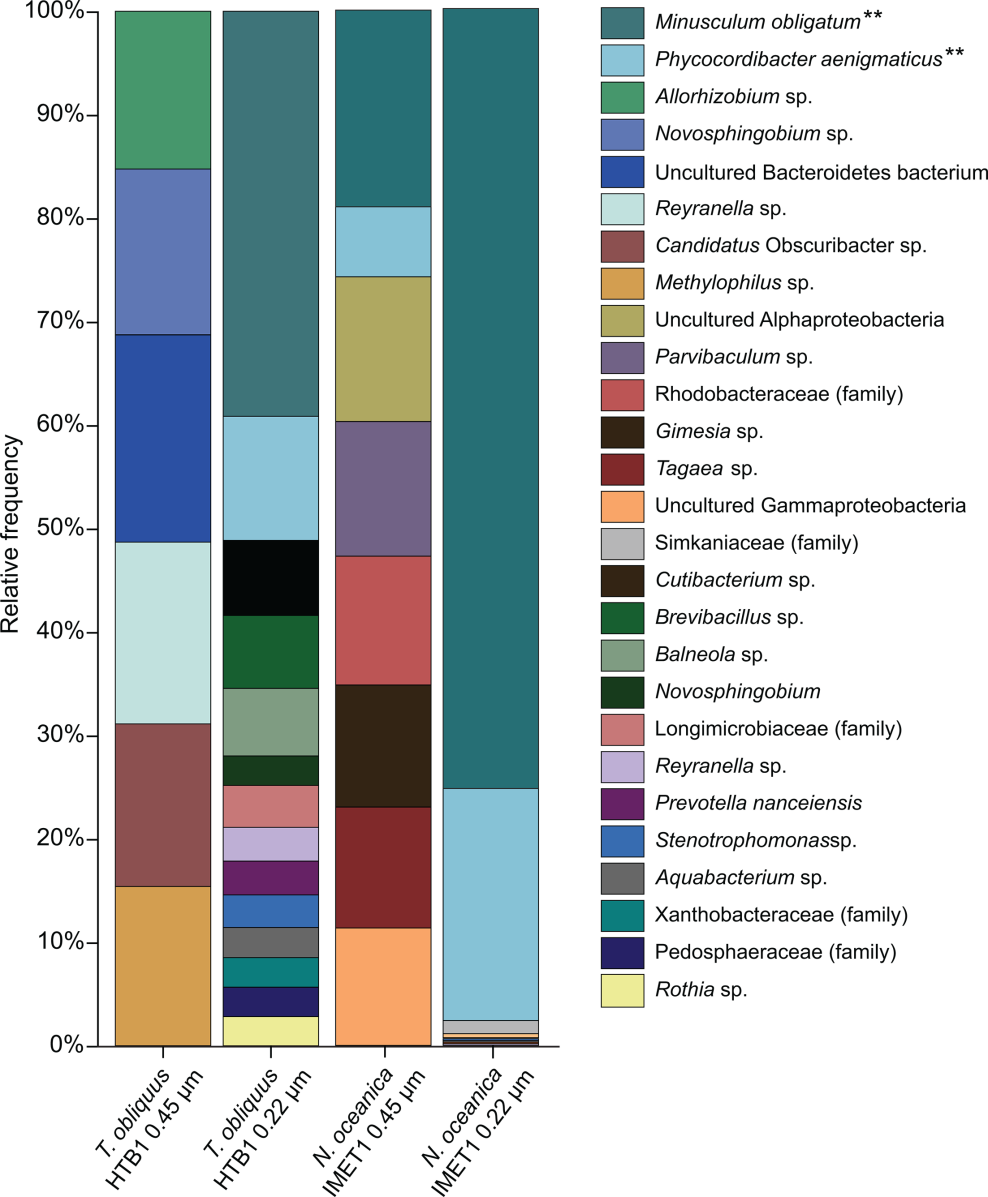
Relative abundance plots of closely associated prokaryotic community (0.45 µm) and free-living prokaryotic community (0.22 µm) of *T. obliquus* HTB1 and *N. oceanica* IMET1 through analysis of the V3/V4 region of the 16S rRNA gene. Both microalgae were grown at the 500 L scale with air enriched with 5% CO_2_. ** = novel Patescibacteria discovered in this study. Chloroplast 16S rRNA gene sequences from *T. obliquus* and *N. oceanica* have been removed bioinformatically.

Two microalgal strains—*N*. *oceanica* strain IMET1 and *T. obliquus* strain HTB1—were grown with simulated flue gas containing 5–10% CO_2_ in 1 L photobioreactors at laboratory scale and with boiler flue gas containing 5% CO_2_ in 500 L photobioreactors at pilot scale at Baltimore’s Back River Waste Water Treatment Plant in an effort to capture flue gas from the plant. Details regarding experimental methods and microalgal growth data can be found in Text S1. To analyze the bacterial communities associated with each microalga, bacteria were collected from robust microalgal cultures (maintained for over 30 days with >30 g/m^2^/day biomass productivity) and separated into two fractions. Symbionts considered to be “closely associated” with the microalgae were captured on 0.45 µm filters (along with the algae), and symbionts considered as “free-living” were concentrated from the filtrate that passed through the 0.45 µm filters and were subsequently captured on 0.22 µm filters. Within the 0.45 µm fraction of both microalgae, the bacterial communities have a high evenness, and *Allorhizobium* sp., *Novospingobium* sp., *Reyranella* sp., *Candidatus* Obscuribacter sp., *Methylophilus* sp., and an uncultured member of the Bacteriodetes phylum were found to be associated with *T. obliquus* HTB1 (Fig. 1). Within the *N. oceanica* IMET1 microbiome at the 0.45 µm fraction, novel Patescibacteria, *M. obligatum* gen. nov. sp. nov., and *P. aenigmaticus* gen. nov. sp. nov. were found along with *Parvibaculum* sp., *Gimesia* sp., *Tagaea* sp., and members of the Alphaproteobacteria and Gammaproteobacteria phyla and Rhodobacteriaceae family (Fig. 1). At the 0.22 µm fraction, *M. obligatum* and *P. aenigmaticus* became dominant, making up >50% relative abundance in the *T. obliquus* cultures and >95% relative abundance in the *N. oceanica* cultures. Bacterial communities were also analyzed from *T. obliquus* cultures grown in laboratory culture at the 1 L scale with ambient air and 10% CO_2_ (Fig. S1 and S2). One bacterium, *Allorhizobium* sp., was found at both the 500 L and 1 L scales (Fig. 1; Fig. S1). Others with high relative abundance found at the 1 L scale were *Algoriphagus aquaeductus*, *Erythrobacter* sp., and *Lacibacter cauensis* (Fig. S1).

### Genomic details and phylogenetic analysis of seven recovered MAGs

**Fig 2:**
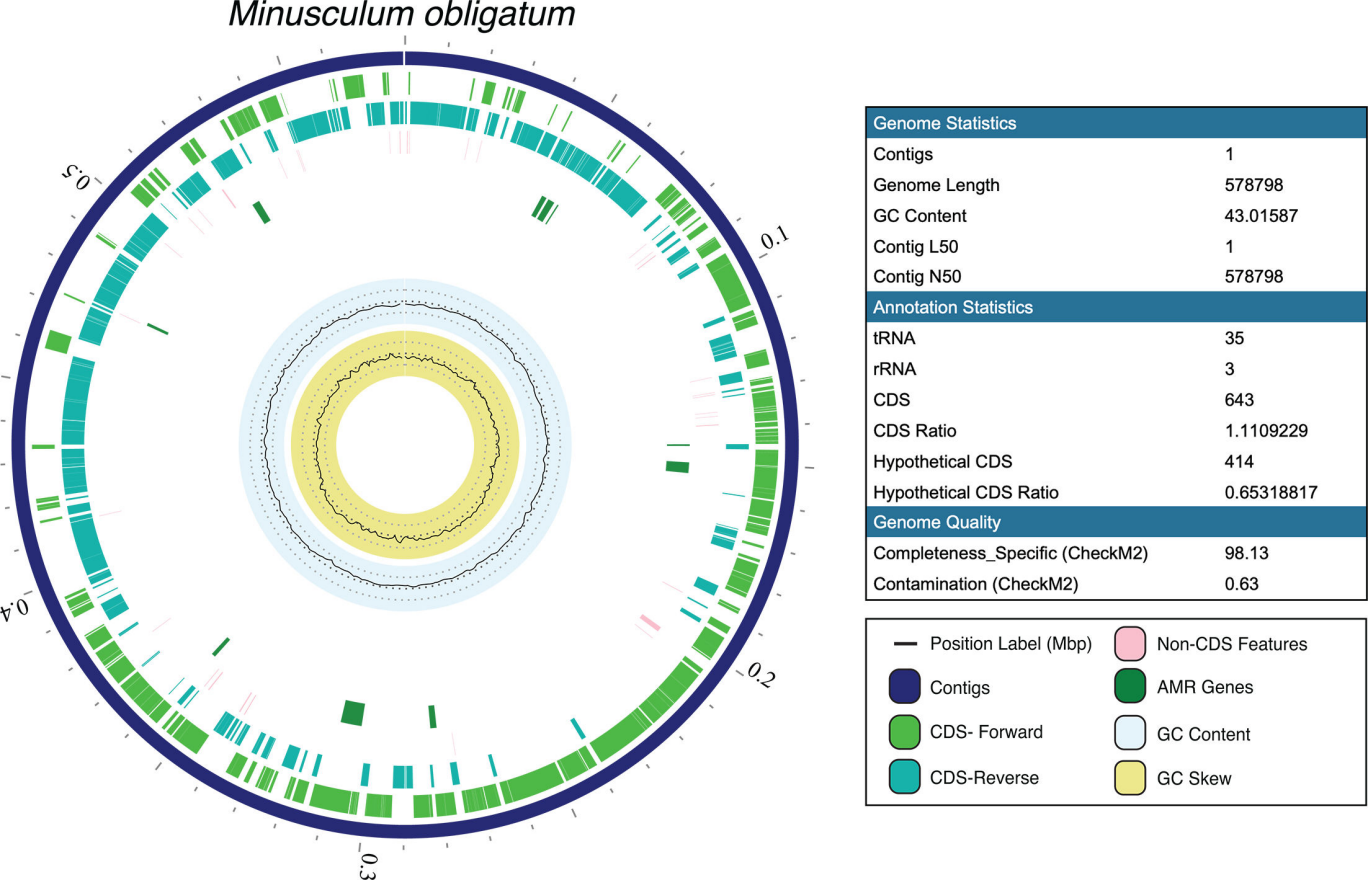
Circular genome view of *M. obligatum*, a MAG recovered from cultures of *N. oceanica* IMET1 and *T. obliquus* HTB1. Genome visualization modified from the BV-BRC. Genome statistics, annotation statistics, and genome completeness and quality are featured to the right.

**Fig 3:**
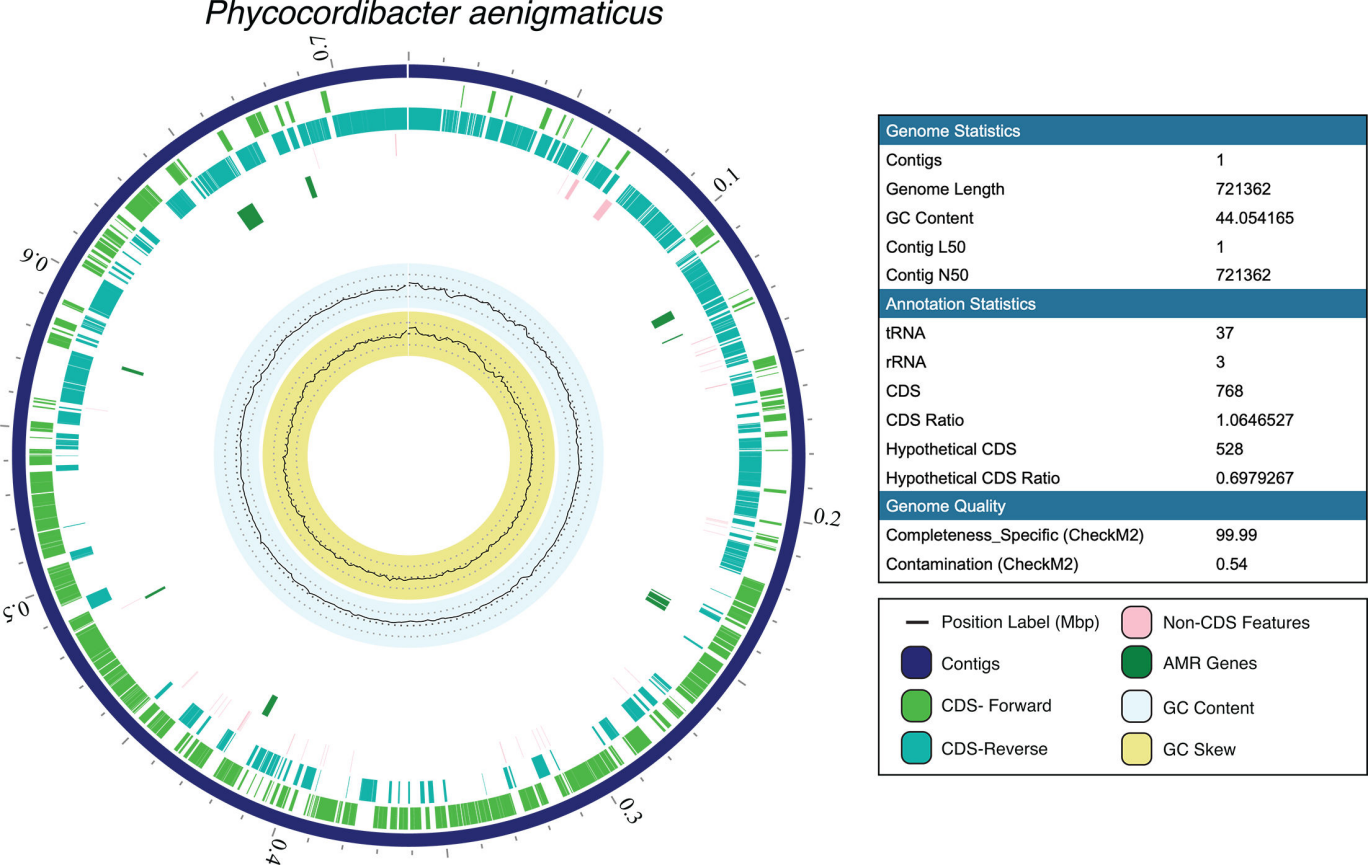
Circular genome view of *P. aenigmaticus*, a MAG recovered from cultures of *N. oceanica* IMET1 and *T. obliquus* HTB1. Genome visualization modified from the BV-BRC. Genome statistics, annotation statistics, and genome completeness and quality are featured to the right.

Samples were chosen for metagenomic sequencing with the aim of recovering metagenomes from the two novel Patescibacteria of interest, *M. obligatum* and *P. aenigmaticus*, as well as other bacteria within the *N. oceanica* and *T. obliquus* microbiomes. Sample “IMET1 0.22 µm” was chosen due to *M. obligatum* and *P. aenigmaticus* making up >95% of relative abundance (Fig. 1). Samples “air day 14” from the 0.45 µm fraction as well as “CO_2_ day 10” and “CO_2_ day 14” from the 0.22 µm fraction were chosen with the aim of recovering metagenomes from a diverse set of bacteria from *T. obliquus* cultures (Fig. S1). Finally, one other sample was chosen from *N. oceanica* cultures bubbled with 10% CO_2_ at the 1 L scale with the aim of recovering metagenomes associated with this alga (49). In all, seven MAGs were recovered: the two dominant Patescibacteria from both algal cultures at 500 L (Fig. 2 and 3), two additional bacteria from *N. oceanica* cultures (Fig. S3 and S4), and three from *T. obliquus* cultures (Fig. S5 to S7). While the two Patescibacteria are the focus of this study due to their novelty and enigmatic metabolism, the five additional MAGs provide a basis for directly comparing the genome characteristics of neighboring bacterial symbionts within our system.

Details regarding sequencing rounds, including total bases called, reads recovered, and quality scores, are shown in Tables S1 and S2. Genome characteristics of all seven MAGs are shown in Table 1. *M. obligatum* consists of a single circular chromosome containing 578,798 bp and a GC content of 43% (Fig. 2). Completeness and contamination using CheckM2 were measured as 98.13% and 0.63%, respectively (Table 1). In total, 643 protein-coding sequences (CDS) were identified, with 414 (64%) of the predicted proteins having no detectable sequence similarity to other proteins in the SEED database used by RASTtk and were therefore annotated as “hypothetical proteins” (Fig. S8) (50). *P. aenigmaticus* consists of a single circular chromosome containing 721,362 bp and a GC content of 44.1% (Fig. 3). Completeness and contamination were measured as 99.99% and 0.54%, respectively (Table 1). In total, 768 CDS were identified, with 528 (69%) as hypothetical proteins (Fig. S9).

**TABLE 1:** Genome characteristics of the MAGs of this study^*a*^

Parameter	*M. obligatum*	*P. aenigmaticus*	*Gracilimonas sediminicola*	*Microcella pacifica*	*A. aquaeductus*	*Erythrobacter* sp.	*Allorhizobium* sp.
Accession no.	CP174360	CP174359	CP175953	JBJINZ000000000	JBJINY000000000	JBJIOB000000000	JBJIOA000000000
Genome size (bp)	578,798	721,362	3,629,614	2,818,534	5,282,570	3,211,232	5,109,754
Contig count	1	1	1	3	94	56	57
Coverage	5,609	380	140	85	145	16	15
Completeness (%)	98.13	99.99	99.87	81.33	94.45	99.31	95.74
Contamination (%)	0.63	0.54	0.25	1.32	5.47	1.64	2.09
GC content (%)	43.02	44.05	44.04	69.13	44.31	63.63	60.62
N50	578,798	721,362	3,629,614	2,792,760	134,053	121,283	168,988
CDS	645	768	3,328	3,078	5,213	3,218	5,255
L50	1	1	1	1	11	10	10
Ribosomal RNAs	5S (70%), 16S, and 23S	5S (55%), 16S, and 23S	5S, 16S, and 23S	5S, 16S, and 23S	5S, 16S, and 23S	5S, 16S, and 23S	5S, 16S, and 23S
tRNA count	35	37	42	45	52	45	52
Algal host	*T. obliquus* and *N. oceanica*	*T. obliquus* and *N. oceanica*	*N. oceanica*	*N. oceanica*	*T. obliquus*	*T. obliquus*	*T. obliquus*

^*a*^ Completeness and contamination estimates have been calculated with CheckM2 and the Completeness_specific parameter, which applies a lineage-specific set of single-copy marker genes tailored to the genome’s taxonomic group. Abbreviations: bp, base pairs; CDS, coding DNA sequences; N50, the length of the shortest contig (in bp) at which half of the entire genome assembly is contained in contigs of this length or longer; L50, minimum number of contigs that make up ≥50% of the total assembled genome length.

Both MAGs encode for one 23S ribosomal RNA, one 16S ribosomal RNA (*M. obligatum*: 1,446 bp; *P. aenigmaticus*: 1,465 bp), and one partial 5S ribosomal RNA (*M. obligatum*: 77% and *P. aenigmaticus*: 55%). The genomes have 35 and 37 predicted tRNAs (*M. obligatum* and *P. aenigmaticus*, respectively), encoding for all amino acids except for asparagine. The 16S rRNA gene sequences extracted from the genomes have 100% identity with *M. obligatum* and *P. aenigmaticus* 16S rRNA gene sequences from the microbiome analysis (Fig. 1). Both MAGs were characterized as high quality by BV-BRC, MiGA, and according to the MIMAG standards: completion >90%; contamination <5%; presence of the 23S, 16S, and 5S rRNA genes and at least 18 tRNAs (51).

**Fig 4:**
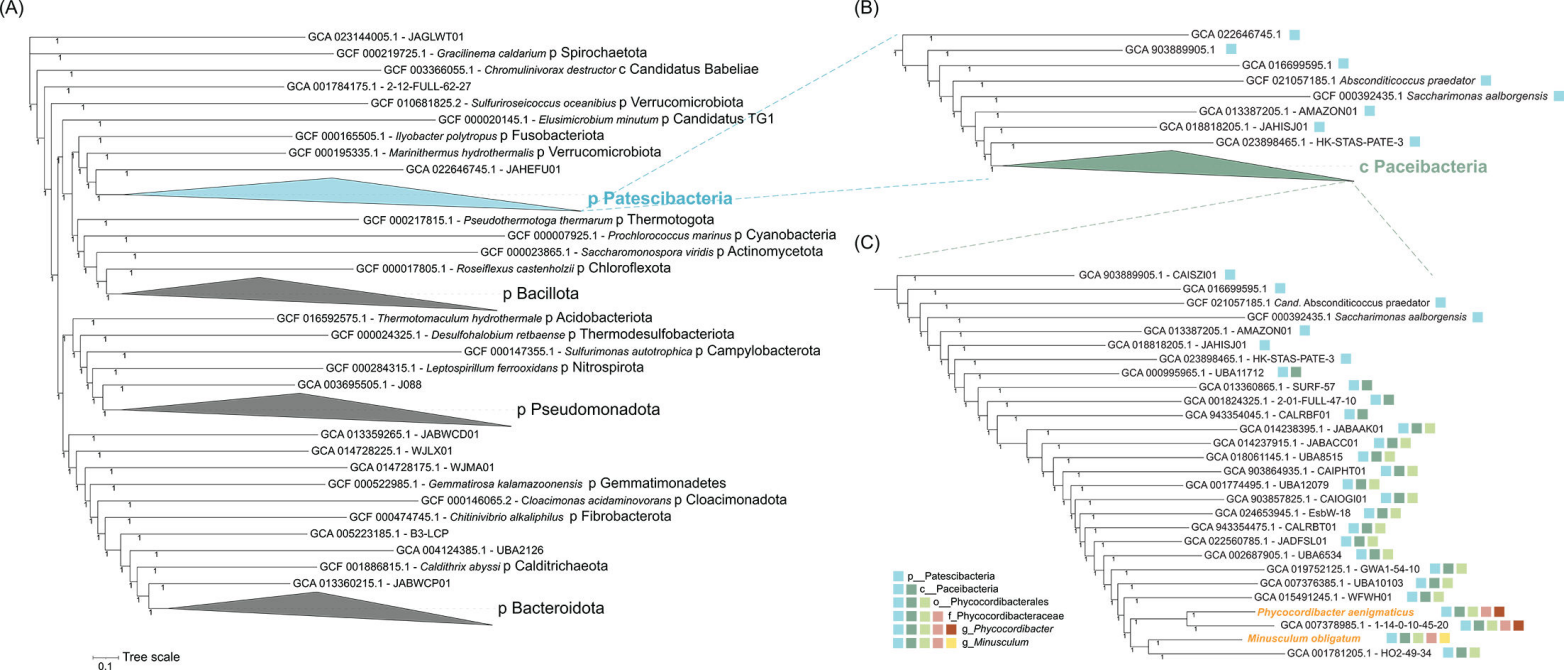
Phylogenetic tree visualizing alignment of 120 concatenated universal bacterial genes generated by GTDB-Tk at the (**A**) phylum level, (**B**) class level, and (**C**) subsequent taxonomic ranks. Bootstrap support values are shown at branch nodes, and multiple members of strongly supported, monophyletic bacterial phyla were collapsed and shown by triangle using iTOL. Light blue shapes denote members of the Patescibacteria phylum, and dark green shapes denote members of the Paceibacteria class. Light green squares denote members belonging to the order Phycocordibacterales ord. nov. Pink squares denote members belonging to the family Phycocordibacteraceae fam. nov. Red squares denote two members belonging to the genus *Phycocordibacter*, and the yellow square denotes one member of the genus *Minusculum*. The collapsed blue triangle in panel **A** is the tree in panel **B**. Furthermore, the collapsed green triangle in panel **B** is the tree in panel **C**.

Phylogenetic trees were constructed to elucidate the potential taxonomic placement of both MAGs. TYGS was used to create full-length 16S rRNA gene trees only with neighbors that have been taxonomically classified (Fig. S10 and S11). However, when working with highly unclassified lineages, it is more advantageous to construct phylogeny with all available taxa, including both formally named bacteria and those given alphanumeric codes. Analysis using an alignment of the full-length 16S rRNA gene shows that the majority of the nearest relatives of *P. aenigmaticus* and *M. obligatum* are classified as “uncultured bacterium clones” and originate from various environments across the world (Tables 2 and 3). A phylogenetic tree constructed with 120 concatenated single-copy universal bacterial genes (bac120) using the GTDB-Tk database provides contextual placement, showing that the two MAGs clearly fall within the Patescibacteria phylum (Fig. 4A) and Paceibacteria class (Fig. 4B).

**TABLE 2:** Nearest neighbors of *M. obligatum* based on BLASTn alignment of the full-length 16S rRNA gene against the NCBI nucleotide database

Nearest neighbor	Accession no.	Query coverage	Percent identity	Environment	Location	Publication status
Uncultured bacterium clone 9M4I 035	JQ287183	100%	92.52%	Deep-sea vent	East Pacific Rise (EPR) near 9°50' N	(52)
Uncultured bacterium clone EV818SWSAP45	DQ337078	100%	87.30%	Subsurface water	Kalahari Shield, South Africa	Unpublished
Uncultured bacterium clone FCPS649	EF516850	100%	86.64%	Soil	Angelo Coast Range Reserve	(53)
Uncultured candidate division OD1 bacterium clone CLN O	KC990422	97%	86.57%	Groundwater	Northwest Colorado	(15)
Uncultured bacterium clone 3M33 009	JQ287225	100%	87.19%	Deep-sea vent	EPR near 9°50' N	(52)
Uncultured bacterium clone TS-33	FJ535317	100%	87.67%	Submarine volcanoes	Kermadec Arc	(54)
Uncultured bacterium clone YCsloP8F1	HE602782	97%	87.11%	Cave walls	Czechia, Spain, and Slovenia	(55)
Uncultured bacterium clone HK31-4-50-1	KX163483	97%	86.90%	Deep basaltic aquifer	Unlisted	Unpublished
Uncultured bacterium clone N1CA0	FQ658793	100%	87.02%	Soil	Unlisted	Unpublished
Uncultured bacterium clone YHY25	GU305806	100%	86.87%	Lake during algal bloom	YangHe reservoir; MinYun reservoir	Unpublished
Uncultured bacterium clone Kili	KX771251	100%	87.34%	Periglacial environment	Mount Kilimanjaro	Unpublished
*Candidatus* Sonnebornia yantaiensis clone YTParaBac1	KC495060 - KC495063	100%	86.80%	Intracellular bacteria of *Paramecium bursaria*		(56)
Uncultured bacterium clone SICT46	LN571156	100%	86.70%	Leaf-cutter ant refuse dump	Unlisted	Unpublished
*Candidatus* Nomurabacteria bacterium isolate HK-STAS-PATE-23	CP060228	100%	85.55%	Activated sludge	Hong Kong	Unpublished
*Candidatus* Kaiserbacteria bacterium isolate Damh_18-Q3-R51-60_MAXAC.235_fly chromosome	CP064968	99%	85.40%	Activated sludge	Unlisted	Unpublished
*Candidatus* Pacebacteria bacterium	OY282360	98%	84.02%	Sponge metagenome	Unlisted	Unpublished

**TABLE 3:** Nearest neighbors of *P. aenigmaticus* based on BLASTn alignment of the full-length 16S rRNA gene against the NCBI nucleotide database

Nearest neighbor	Accession no.	Query coverage	Percent identity	Environment	Location	Publication status
Uncultured bacterium clone T60-An-20C-53	JX105612	100%	92.54%	Fish aquaria	Greece	(57)
Uncultured bacterium clone FD02	AB354611	99%	91.64%	Calcite travertine	Unlisted	Unpublished
Uncultured bacterium clone A69	KJ817567	100%	86.92%	Wetland	Ebinur Lake	Unpublished
Uncultured bacterium clone T0-An-20C-25	JX105530	100%	85.45%	Fish aquaria	Greece	(57)
Uncultured bacterium clone BJGMM-3s-374	JQ801042	99%	86.10%	Soil	Yellow River Delta	Unpublished
Uncultured organism clone SBZI 7733	JN525923	100%	86.09%	Hypersaline microbial mat	Guerrero Negro	(58)
Uncultured bacterium clone SEAB1DH091	KC432348	100%	85.16%	Constructed wetland	Mahdia City, Tunisia	(59)
Uncultured bacterium clone SICX85	LN572061	100%	85.24%	Leaf-cutter ant refuse dumps	Unlisted	Unpublished
Uncultured bacterium clone SSB0101-01	JN397923	100%	84.84%	Spring pits	Jing-Mei River	Unpublished
Uncultured bacterium clone Iron-r	LN870937	100%	84.64%	Fe-rich microbial mats	Unlisted	Unpublished
*Candidatus* Nomurabacteria bacterium isolate HK-STAS-PATE-23	CP060228	100%	85.55%	Activated sludge	Hong Kong	Unpublished
*Candidatus* Kaiserbacteria bacterium isolate Damh_18-Q3-R51-60_MAXAC.235_fly chromosome	CP064968	99%	85.40%	Activated sludge	Unlisted	Unpublished
*Candidatus* Pacebacteria bacterium	OY282360	98%	84.02%	Sponge metagenome	Unlisted	Unpublished

### Formal proposal of *M. obligatum* gen. nov. sp. nov., *P. aenigmaticus* gen. nov. sp. nov., and associated higher taxonomic ranks

We formally propose the following taxonomic names for two novel species, two novel genera, one novel family, and one novel order: *M. obligatum* gen. nov. sp. nov., *P. aenigmaticus* gen. nov. sp. nov., Phycocordibacteraceae fam. nov., and Phycocordibacterales ord. nov. (Table 4). The bac120 tree classified the two MAGs as having belonged to the same phylum (Patescibacteria), same class (Paceibacteria), same order (Phycocordibacterales, replacing the order placeholder o__UBA9973), same family (Phycocordibacteraceae, replacing the family placeholder f__UBA2103), and two distinct genera (*Phycocordibacter*, replacing the genus placeholder g__UBA2103; *Minusculum*, replacing the genus placeholder g__1-14-0-10-45-20; Fig. 4C and Table 4). Data from MiGA reflecting taxonomic novelty (AAI%), dDDH values provided by the TYGS, and RED scores using GTDB-Tk support this proposal of novelty that has been validated by the Seqcode (seqco.de/r:ywe1blo2) (39) (Table 5). The closest relatives found by MiGA for *P. aenigmaticus* in the TypeMat database were *Ammonifex degensii* KC4 (37.03% AAI); for *M. obligatum, Pueribacillus theae* GCF (36.75% AAI) was the closest relative (Table 5). TYGS assigned *Chlamydia psittaci* 6BC as the closest subject strain to *P. aenigmaticus* with a highest dDDH of 13.2% (Table 5; Fig. S11) and *Peribacter riflensis* to *M. obligatum* with a dDDH of 16.9% (Table 5; Fig. S10). RED scores using the bac120 alignment (Fig. 4) were 0.92162 for *P. aenigmaticus* and 0.93255 for *M. obligatum*.

**TABLE 4:** List of proposed names and their current placeholders

Proposed type MAG accession	Proposed species name	Current name of order	Proposed order	Current name of family	Proposed family	Current name of genus	Proposed genus
CP174359	*P. aenigmaticus*	o__UBA9973	Phycocordibacterales	f__UBA2103	Phycocordibacteraceae	g__1-14-0-10-45-20	*Phycocordibacter*
CP174360	*M. obligatum*	o__UBA9973	Phycocordibacterales	f__UBA2103	Phycocordibacteraceae	g__UBA2103	*Minusculum*

**TABLE 5:** Protologs of the MAGs of this study as suggested by Chuvochina et al. (40) and the proposed minimal standards set forth by Riesco and Trujillo (39)^*a*^

Parameter	*M. obligatum* gen. nov. sp. nov.	*P. aenigmaticus* gen. nov. sp. nov.	*Gracilimonas sediminicola*	*Microcella pacifica*	*A. aquaeductus*	*Erythrobacter* sp.	*Allorhizobium* sp.
Accession no.	CP174360	CP174359	CP175953	JBJINZ000000000	JBJINY000000000	JBJIOB000000000	JBJIOA000000000
Species name	*M. obligatum*	*P. aenigmaticus*	--	--	--	--	--
Genus name	*Minusculum*	*Phycocordibacter*	--	--	--	--	--
Specific epithet	*obligatum*	*aenigmaticus*	--	--	--	--	--
Genus etymology	neut. n. *Minusculum*, rather small, referring to the small genome size	Gr. neut. n. phyco, meaning seaweed or algae; L. neut. n. cor, from *cordis*, meaning central or essential; N.L. masc. n. bacter, referring to a bacterium; N.L. masc. n. *Phycocordibacter*, a core bacterium of a microalgal culture		--	--	--	--
Species etymology	neut. adj. *obligatum*, to bind, oblige, or require	masc. adj. *aenigmaticus*, enigmatic or obscure	--	--	--	--	--
Genus status	gen. nov.	gen. nov.	--	--	--	--	--
Species status	sp. nov.	sp. nov.	--	--	--	--	--
Pairwise ANI (%)	N/A	N/A	99.48	97.71	98.06	--	--
AAI (%)^*b*^	36.75%	37.03%	--	--	--	--	--
dDDH (%)^*c*^	16.9%	13.2%	--	--	--	--	--
RED score	0.93255	0.92162					
Taxonomic novelty (*P*-value ≤ 0.01 suggests novelty**)**^*d*^	Phylum = 0.123 Class = 0.00622 Order = 0.000576 Family = 0.00025 Genus = 0.000246	Phylum = 0.143 Class = 0.0101 Order = 0.00133 Family = 0.000379 Genus = 0.000246	--	--	--	--	--
Designation of the type MAG	CP174360	CP174359	GCA_024320785.1	GCF_009371975.2	GCF_003253485.1	No type designated	GCF_013318015.2
Status of MAG	Draft	Draft	Draft	Draft	Draft	Draft	Draft
Genome size (bp)	578,798	721,362	3,629,614	2,818,534	5,282,570	3,211,232	5,109,754
GC content (%)	43.02	44.05	44.04	69.13	44.31	63.63	60.62
Completeness (%)	98.13	99.99	99.87	81.33	94.45	99.31	95.74
Contamination (%)	0.63	0.54	0.25	1.32	5.47	1.64	2.09
Contig count	1	1	1	3	94	56	57
N50	578,798	721,362	3,629,614	2,792,760	134,053	121,283	168,988
L50	1	1	1	1	11	10	10
Coverage	5,609	380	140	85	145	16	15
Country of origin	USA	USA	USA	USA	USA	USA	USA
Region of origin	Baltimore, MD	Baltimore, MD	Baltimore, MD	Baltimore, MD	Baltimore, MD	Baltimore, MD	Baltimore, MD
Source of sample	Microalgal culture	Microalgal culture	Microalgal culture	Microalgal culture	Microalgal culture	Microalgal culture	Microalgal culture
Algal host name(s)	*N. oceanica* and *T. obliquus*	*N. oceanica* and *T. obliquus*	*N. oceanica*	*N. oceanica*	*T. obliquus*	*T. obliquus*	*T. obliquus*
Sampling date (yr-mo-day)	2022-05-01	2023-01-20	2022-05-01	2022-05-01	2021-12-12	2021-12-12	2021-12-12
Geographic location	Institute of Marine and Environmental Technology	HyTek Bio LLC	Institute of Marine and Environmental Technology	Institute of Marine and Environmental Technology	Institute of Marine and Environmental Technology	Institute of Marine and Environmental Technology	Institute of Marine and Environmental Technology
Latitude	39.2860027 N	39.298342 N	39.2860027 N	39.2860027 N	39.2860027 N	39.2860027 N	39.2860027 N
Longitude	76.6056885 W	76.496257 W	76.6056885 W	76.6056885 W	76.6056885 W	76.6056885 W	76.6056885 W
Assembly	Three samples	Three samples	One sample	One sample	One sample	One sample	One sample
Sequencing platform	Oxford Nanopore GridION	Oxford Nanopore GridION	Oxford Nanopore GridION	Oxford Nanopore GridION	Oxford Nanopore GridION	Oxford Nanopore GridION	Oxford Nanopore GridION
Assembly program	Flye v2.9.2	Flye v2.9.2	Flye v2.9.2	Flye v2.9.2	Flye v2.9.2	Flye v2.9.2	Flye v2.9.2
Binning program	MaxBin2 v2.2.4	MaxBin2 v2.2.4	MaxBin2 v2.2.4	MaxBin2 v2.2.4	CONCOCT v1.1	CONCOCT v1.1	CONCOCT v1.1
Predicted proteins	641	749	3,243	3,009	4,770	3,143	5,052
Ribosomal RNAs	5S (70%), 16S, and 23S	5S (55%), 16S, and 23S	5S, 16S, and 23S	5S, 16S, and 23S	5S, 16S, and 23S	5S, 16S, and 23S	5S, 16S, and 23S
tRNA count	35	36	42	47	52	45	52
BioSample	SAMN44533063	SAMN44533074	SAMN44533075	SAMN44533076	SAMN44533081	SAMN44533102	SAMN44533082

^*a*^ “--” denotes non-applicability with certain rows regarding taxonomic novelty.

^*b*^ AAI% of closest relatives found by MiGA using the TypeMat database.

^*c*^ dDDH formula d4 recommended by the TYGS.

^*d*^ Taxonomic novelty determined by MiGA using the TypeMat database.

### Description of *M. obligatum*

We propose *M. obligatum* gen. nov. sp. nov. with *Minusculum* replacing the alphanumeric genus place holder g__1-14-0-10-45-20. Species etymology: *M. obligatum*—syllabification o.bli.ga′tum—from Latin neut. adj. *obligatum*, to bind, oblige, or require. Genus etymology: *Minusculum*—Mi.nu.scu.lum—Latin neut. noun, meaning rather small, referring to the genome size. Thus, *M. obligatum* is an obligate symbiont with a highly reduced genome (~600 kbp). A formal protolog has been created for this proposed name in Table 5.

### Description of *P. aenigmaticus*

We propose *P. aenigmaticus* gen. nov. sp. nov. with *Phycocordibacter* replacing the alphanumeric genus place holder g__UBA2103. Species etymology: *P. aenigmaticus*—syllabification ae.nig.ma′ti.cus—from the Latin masculine adjective *aenigmaticus*, meaning enigmatic or obscure (Table 5). Genus etymology: *Phycocordibacter*—Phy.co.cor.di.bacter—is split into three distinct morphemes: Greek neuter noun phyco, from *phykos*, meaning seaweed or algae; L. neut. n. cor, from *cordis*, meaning central or essential; N.L. masc. n. bacter, referring to a bacterium. Thus, N.L. masc. n. *P. aenigmaticus* is an enigmatic and core bacterium of a microalgal culture. The inferred stem, *Phycocordibacter*-, was used to create subsequent family and order level nomenclature.

### Description of Phycocordibacteraceae fam. nov

We propose Phycocordibacteraceae (Phy.co.cor.di.bac.ter.a′ce.ae) to replace family placeholder f__UBA2103. Phycocordibacter is the type genus of the family; -aceae ending to denote a family. Thus, N.L. fem. pl. n. Phycocordibacteraceae, the Phycocordibacter family. *M. obligatum* also falls within this family.

### Description of Phycocordibacterales ord. nov

We propose Phycocordibacterales (Phy.co.cor.di.bac.ter.ales) to replace order placeholder o__UBA9973. Phycocordibacter is the type genus of the order; ‐ales ending to denote an order. Thus, N.L. fem. pl. n. Phycocordibacterales, the Phycocordibacter order. *M. obligatum* also falls within this order.

### Lifestyle predictions

The symbiont classifier, symcla, uses a machine learning-based approach to predict lifestyle based on 56 universal bacterial marker genes found in each MAG, UNI56. Scores assigned by symcla are interpreted as follows:

symcla_score ≤ 0.4 = free-living

0.42 < symcla_score < 1.21 = symbiont (host-associated)

symcla_score ≥ 1.21 = symbiont (intracellular).

**Fig 5:**
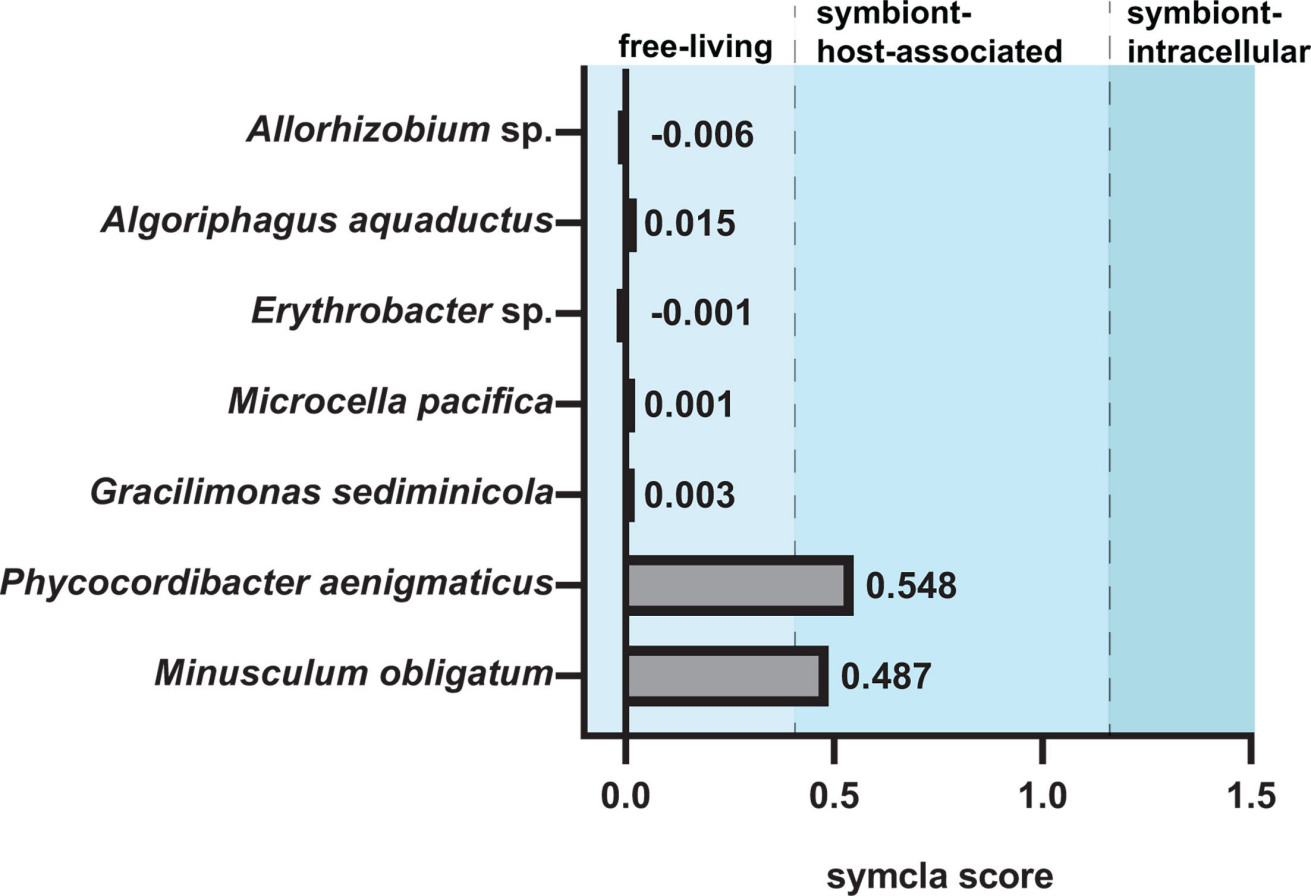
Symbiont scores of each MAG as determined by symcla. Light blue shading denotes a score correlated with free-living bacteria, medium blue denotes host-associated symbionts, and dark blue denotes intracellular symbionts.

*M. obligatum* and *P. aenigmaticus* both scored as being host-associated symbionts (0.487 and 0.548, respectively), and the remaining five classified MAGs scored as being free-living (all ≤0.003; Fig. 5; Table S3). Two bacteria, *Allorhizobium* sp. and *Erythrobacter* sp., scored negative, this being a common occurrence denoting free-living status.

### Core metabolism of *M. obligatum* and *P. aenigmaticus*

**Fig 6:**
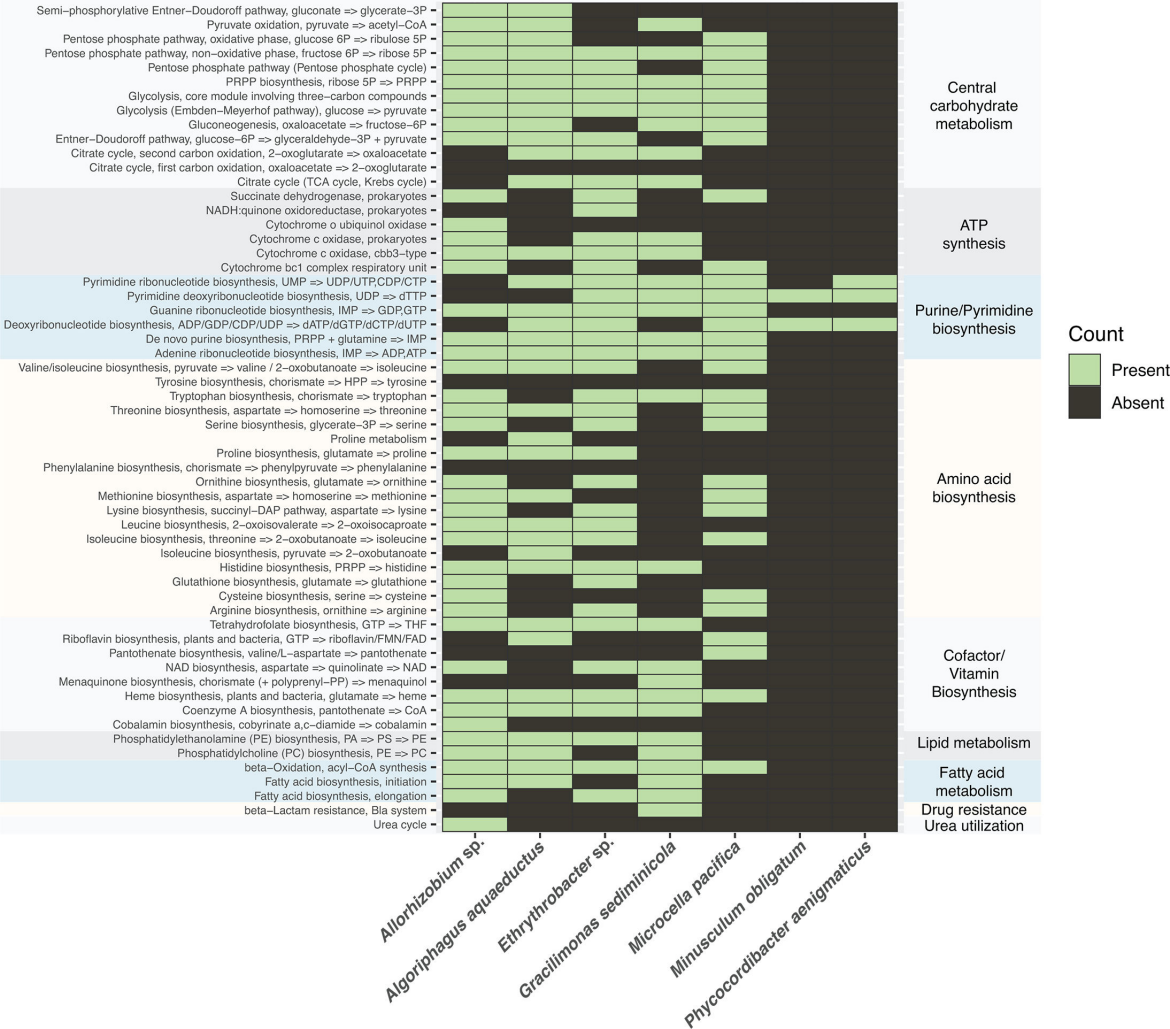
Heatmap (presence or absence) displaying metabolic capacity of all seven MAGs predicted with METABOLIC-G (48) querying annotated proteins with the KEGG database. A complete pathway was labeled as present if 75% or more of the steps involved in that pathway were found to be present.

The metabolic capacity of all seven MAGs was predicted with METABOLIC-G, which queries annotated proteins of each MAG against HMM databases (KEGG KOfam, Pfam, TIGRfam, and custom HMMs), resulting in KEGG modules (Fig. 6) and custom HMM hit profile (Table S12) (48, 60). Figure 6 shows the absence of complete carbohydrate metabolism pathways in both *M. obligatum* and *P. aenigmaticus*, implying neither bacterium can utilize or produce sugars through central carbon metabolism. Both cannot fully oxidize organic carbon to CO_2_ for energy generation through the TCA cycle, and the lack of ATP synthesis genes indicates that they rely on alternative electron donors for metabolic processes. Both bacteria, however, do contain genes for purine and pyrimidine biosynthesis (Fig. 6).

When analyzing individual genes that make up complete pathways for energy production (i.e., glycolysis), we discovered that both *M. obligatum* and *P. aenigmaticus* encode genes within the reductive pentose phosphate pathway (Calvin cycle). *M. obligatum* encodes for 10 out of the 22 genes needed for a complete pathway, and *P. aenigmaticus* encodes for 12 out of 22 (Table 6). The incomplete pathway in both organisms suggests that the Calvin cycle may not function for full carbon fixation but instead for the biosynthesis of reducing power, NADPH. Additionally, *P. aenigmaticus* encodes for *atpA* (V/A-type), one gene of four encoding for Complex V ATP synthase, indicating a potential alternative mode for ATP production (Fig. S12).

**TABLE 6:** Gene counts from major energy-producing pathways annotated with KEGG^*a*^

Species	Genes present				
	Glycolysis (total genes, 14)	Gluconeogenesis (total genes, 7)	PPP (total genes, 13)	TCA cycle (total genes, 16)	Reductive PPP (Calvin cycle) (total genes, 22)
*Allorhizobium* sp.	14	6	13	6	14
*A. aquaeductus*	14	7	13	12	16
*Erythrobacter* sp.	11	5	11	12	14
*Gracilimonas sediminicola*	14	7	9	14	12
*Microcella pacifica*	13	7	12	10	16
*M. obligatum*	3	2	2	0	10
*P. aenigmaticus*	4	2	4	0	12

^*a*^ Total amount of genes in the pathway is displayed under the pathway name with individual genes found within each MAG listed below. If the pathway was 75% complete, it appears as present in Fig. 6. PPP = pentose phosphate pathway.

*P. aenigmaticus* possesses the gene *sdo* (sulfur dioxygenase), suggesting it can oxidize elemental S (S^0^) to sulfite (SO_3_^2−^), but it lacks upstream sulfur oxidization genes (i.e., *FccAB,* flavocytochrome c sulfide dehydrogenase), which would oxidize hydrogen sulfide (Fig. S12). *P. aenigmaticus* also encodes for *nirK*, a nitrite reductase (NO-forming), but lacks all other nitrogen reduction genes (i.e., *nirS* and *nosZ*; Fig. S12). Instead of full denitrification to N_2_ gas, the bacterium can only reduce nitrite (NO_2_^−^) to nitric oxide (NO). *M. obligatum*, on the other hand, encodes for no genes involved in S and N metabolism.

## DISCUSSION

### Bacteria associated with the microalgae *T. obliquus*

The most abundant bacterial taxon associated with *T. obliquus* was *A. aquaeductus*, a genus that has been found previously to be associated with several strains of microalgae, including *N. oceanica* (61) (Fig. S1). *Erythrobacter* sp. (formally classified as *Porphyrobacter* spp.) was previously found in our laboratory to be the most dominant organism associated with *T. obliquus* cultures bubbled with flue gas (comprising 56% of 16S rRNA gene sequences) and ambient air (85%) (62). *Allorhizobium* sp. was the only bacterium found at both the laboratory scale (1 L; Fig. S1) and pilot scale (500 L; Fig. 1). It is a urease-producing bacterium that is of potential interest within carbon capture technologies due to its potential ability to convert atmospheric CO_2_ to calcium carbonate through microbially induced calcium carbonate precipitation (63). At the 500 L scale, notable members of the *T. obliquus* microbiome at the 0.45 µm level include *Novosphingobium* sp., a bacterium that has previously shown to promote the growth of cyanobacteria (64).

### Unexpected and unprecedented dominance of novel Patescibacteria within microalgal cultures

Patescibacteria have rarely been reported to be associated with microalgae apart from microbial fuel cells with algal-film cathodes (65) and algal-bacterial biofilms in wastewater treatment facilities (66–68). The latter is to be expected as Patescibacteria are commonly found within wastewater treatment plants (22). For example, a Patescibacteria (order Saccharimonadales) emerged as the dominant taxa in activated sludge that was aerated by a separated microalgal biofilm reactor. Although this bacterium was not in contact with the microalgae (*T. obliquus*), the bacterial community clearly shifted after 10 days with Patescibacteria becoming the most abundant phylum at 44% relative abundance (69). It is likely that other diverse members of Patescibacteria have been identified within algal microbiomes, although they are often present at lower relative abundances and remain unclassified taxonomically. For example, *Ca.* Sonnebornia yantaiensis listed in Table 2 was discovered living inside of the ciliate *Paramecium bursaria* (56). *Ca.* Sonnebornia yantaiensis seems to form a symbiotic partnership by clustering with the alga *Chlorella* sp., a fellow endosymbiont of *P. bursaria*. Additionally, there have been reports of an “uncultured marine bacterium” with phylogenetic affiliations to *Ca*. Gracilibacteria associated with marine macroalgae and an “uncultured bacterium” with phylogenetic affiliations to *Ca*. Peregrinibacteria associated with diatom blooms (70). Although it is common to find uncultivated bacteria within microbiome data (71), it was unexpected that two Patescibacteria were found in high relative abundance repeatedly throughout multiple rounds of our experiments and at multiple scales. It was their high relative abundance that provided us with a unique opportunity to sequence their metagenomes with high coverage and establish type strains for two novel genera, one novel family, and one novel order.

Researchers working with Patescibacteria are currently at an exciting frontier, although working with a phylum in which the majority of lower taxonomic ranks remain unnamed can pose challenges (10). Both *M. obligatum* and *P. aenigmaticus* have no closely related MAGs that have been formally named and published (Tables 2 and 3), even at the order level (Fig. 4B). The nearest cultured relatives are *Saccharimonas aalborgensis* and *Ca.* Absconditicoccus praedator (Fig. 4C), and both belong to different classes than that of *M. obligatum* and *P. aenigmaticus*. This fact can make 16S rRNA gene marker trees and concatenated gene phylogenomic trees (Fig. 4) not as informative as other trees that display more classified lineages. As more classes, orders, and lower ranks of Patescibacteria are designated type strains and given formal names, both gene marker and concatenated gene trees will become more informative. In the current period of time during which most members of Patescibacteria have alphanumeric codes instead of names, it is largely recommended to construct a phylogeny with 120 ubiquitous single-copy marker genes, use continuously updated (and publicly available) taxonomic resources such as the TYGS and GTDB, and propose names in collaboration with the SeqCode (10, 39, 44, 72, 73).

### Genome reduction and a lack of essential genes: familiar features of Patescibacteria

Through 16S rRNA gene analysis, Patescibacteria have been found to represent approximately 50% of bacterial lineages of phylum-level evolutionary distance (19). However, this could be due to the atypical evolution of ribosomal proteins, as Parks et al. (74) reported that Patescibacteria comprise approx. 25% of phylum-level lineages when phylogeny was constructed based on 120 concatenated proteins (74); others have reported lower than 25% (75). Two seemingly constant features of Patescibacteria are small genomes and ultra-small cell sizes (17, 76). For example, several Patescibacteria were reported in groundwater samples that passed through 0.22 µm filters with cell sizes of 0.009 ± 0.002  µm^3^ (15). In this study, *M. obligatum* and *P. aenigmaticus* were found in high relative abundance within the 0.22 µm fraction of the 500 L bioreactor, as well as within the 0.45 µm fraction in *N. oceanica* cultures (Fig. 1). There is a likelihood that both *M. obligatum* and *P. aenigmaticus* are symbionts of other microalgae-associated bacteria rather than of the microalgae themselves, based on their prevalence in the free-living (0.22 µm) fraction rather than in the microalga-associated (0.45 µm) fraction. Symbiosis between Patescibacteria and a bacterial host has been described previously (7). Additionally, two CPR bacteria—a member of Saccharibacteria (formerly known as TM7) and *Vampirococcus lugosii*—are both obligate, parasitic epibionts of the bacteria *Actinomyces odontolyticus* and *Halochromatium* sp., respectively (7, 11). In previous work, we found *M. obligatum* associated with *N. oceanica* at the 1 L culture scale in the closely associated fraction (0.45 µm) in similar relative abundance to the free-living fraction (0.22 µm) (23, 49). We have never found either Patescibacterium solely in the microalgae cell-associated (0.45 µm) fraction, strongly suggesting that the Patescibacteria are symbionts of other bacteria rather than the microalgae.

Consistent with previously reported Patescibacterial genome sizes of <1 Mb, this study recovered reduced genomes of *M. obligatum* (578,798 bp; Fig. 2) and *P. aenigmaticus* (721,362 bp; Fig. 3). Even within highly reduced genomes, much information can be gleaned regarding metabolic function, with “complete” pathways defined in this study as ≥75% of encoded genes for biochemical reactions to accomplish a certain metabolic function (Fig. 6). Genome completeness has been shown to contribute a significant bias on recovered functional signal, and metagenomes can often display incomplete metabolic pathways compared to isolate genomes due to low completeness (48, 77). Thus, we acknowledge that the functional capacities presented in Fig. 6 may be underestimated. This could be particularly true for *Microcella pacifica* (completeness = 81.33%) and *A. aquaeductus* (completeness = 94.45%; Table 1).

### Metabolic reconstruction reveals extreme limitations

Reduced genome sizes lead to another hallmark feature of Patescibacteria, limited metabolic capabilities (13, 76, 78). Our findings are consistent with this trend as neither *M. obligatum* nor *P. aenigmaticus* encode for complete pathways for glycolysis, gluconeogenesis, the pentose phosphate pathway, the TCA cycle, and ATP synthesis (Fig. 6). Taking into consideration the absence of key metabolic genes for energy production along with their symcla scores between 0.42 and 1.21, we surmise *M. obligatum* and *P. aenigmaticus* to both be obligate symbionts of *T. obliquus* HTB1 and *N. oceanica* IMET1, or more likely a neighboring bacterium.

Along with the typical absence of complete pathways for central carbohydrate metabolism, both *M. obligatum* and *P. aenigmaticus* also lack all amino acid biosynthetic pathways (Fig. 6). This finding is striking and in agreement with recent studies, with the exception of that of Haro‐Moreno et al., who reported many amino acid biosynthetic routes found within a collection of Patescibacteria, especially within their recovered MAGs from Lake Baikal (79). One gene of interest encoded by *P. aenigmaticus*, *nirK*, provides some evidence for preserved metabolic function across Patescibacteria as it is found within various lineages and thought not to be acquired by horizontal gene transfer (80, 81). This function could be a strategy for managing nitrite toxicity, especially in the presence of a neighboring organism able to further reduce nitric oxide such as *Allorhizobium* sp. (this study), which encodes for both nitric oxide reductase subunits B and C, *norB* and *norC* (Fig. S12). *P. aenigmaticus* possesses the gene *sdo* (sulfur dioxygenase), suggesting it can oxidize elemental S (S^0^) to sulfite (SO_3_^2−^) but lacks upstream sulfur oxidization genes. Instead, *P. aenigmaticus* likely relies on sulfur within the algal growth medium as a terminal electron acceptor, with the oxidation reaction generating NADPH to support other biosynthetic processes. Three other Patescibacteria have been shown to encode *sdo* as well as other genes involved in sulfate reduction, *sat*, *cysC*, and *cysN*, all three of which were not encoded by the ones in this study (81). However, *cysN* and *cysD* were encoded by four other MAGs recovered from our system, implying there might exist shared redox reactions within bacterial communities, as may be the case with nitrite and nitric oxide reduction.

Without complete pathways encoding for glycolysis, gluconeogenesis, or the TCA cycle, Patescibacteria generally cannot generate energy or biosynthetic intermediates from organic carbon. Moreover, the absence of complete ATP synthesis pathways indicates that oxidative phosphorylation pathways are not utilized to generate energy in these bacteria. The only exception was one gene, *atpA* (V/A-type) of Complex V ATP synthase, encoded by *P. aenigmaticus* (Fig. S12). Instead, *M. obligatum* and *P. aenigmaticus* encode for a part of the reductive pentose phosphate (Calvin cycle) pathway, genes for which have also been found in other Patescibacteria, including Paceibacteria (Table 6) (20, 82). However, the incomplete pathway suggests that it is not functional for complete CO_2_ fixation and metabolic independence. The presence of genes encoding for Calvin cycle machinery, albeit incomplete, suggests their ability to produce NADPH and potentially ribose-5-phosphate nucleotides. Therefore, both are likely obligatory symbionts that depend on essential metabolites from algal hosts, other bacteria within the system, or from scavenging compounds within the algal growth medium and/or phycosphere. This observation aligns with adaptations observed in intracellular symbionts and bacteria with highly reduced genomes (83). We ascertain both bacteria are highly obligate but not endosymbiotic based on their symcla scores (Fig. 5) and ability to pass through a 0.45 µm filter (Fig. 1; Fig. S1). These findings also suggest that they may form an obligate symbiosis with another bacterium present in the algal phycosphere.

### Conclusion

Although Patescibacteria are often reported to be incompletely recovered in amplicon-based data sets (84), the opposite result was found in this study. In fact, 16S rRNA gene sequences of two unclassified Patescibacteria repeatedly had the highest relative abundance in bacterial communities associated with two different species of microalgae. Within the 0.22 µm fraction, the two Patescibacteria made up >50% relative abundance in the *T. obliquus* cultures and >95% relative abundance in the *N. oceanica* cultures. Because of this dominance, we deployed metagenomic sequencing and recovered seven high-quality MAGs, two of which belonged to the Patescibacteria of interest. We then worked closely with the SeqCode to establish taxonomic novelty and assign names for two novel genera, one novel family, and one novel order. Here, we present *M. obligatum* and *P. aenigmaticus*, both with highly reduced genomes and limited metabolic capacities that are consistent with that of other reported Patescibacteria (76, 85). We show that neither bacterium has complete pathways in central carbohydrate metabolism, ATP synthesis, or amino acid biosynthesis. Despite these extreme metabolic deficiencies, we found *M. obligatum* and *P. aenigmaticus* as dominant symbionts across various experimental metrics. They are associated with two ecologically distinct microalgae—one freshwater strain *T. obliquus* HTB1 and one marine strain *N. oceanica* IMET1—at two different scales (1 L and 500 L), within two different filter size fractions (0.45 µm and 0.22 µm) and regardless of whether cultures were bubbled with ambient air or with simulated flue gas containing 5% or 10% CO_2_. They are most likely obligate symbionts of the microalgae or other metabolically active neighboring bacteria, which may explain why they are unable to be cultivated outside of the algal system.

## Data Availability

MAG accession numbers are as follows: *Phycocordibacter aenigmaticus*, CP174359; *Minusculum obligatum*, CP174360; *Gracilimonas sediminicola*, CP175953; *Microcella pacifica*, JBJINZ000000000; *Algoriphagus aquaeductus*, JBJINY000000000; *Allorhizobium* sp., JBJIOA000000000; and *Erythrobacter* sp., JBJIOB000000000. The SeqCode Registry accession is as follows: seqco.de/r:ywe1blo2.

## SUPPLEMENTAL MATERIAL

Supplemental material mbio.01231-25-s0001.pdf Text S1, Figures S1–S12, and Tables S1–S3.

ASM does not own the copyrights to Supplemental Material that may be linked to, or accessed through, an article. The authors have granted ASM a non-exclusive, world-wide license to publish the Supplemental Material files. Please contact the corresponding author directly for reuse.
