# Erratum for Jonas et al., “Two novel Patescibacteria: *Phycocordibacter aenigmaticus* gen. nov. sp. nov. and *Minusculum obligatum* gen. nov. sp. nov., both associated with microalgae optimized for carbon dioxide sequestration from flue gas”

**DOI:** 10.1128/mbio.00599-26

**Published:** 2026-03-20

**Authors:** Lauren Jonas, Yi-Ying Lee, Tsvetan Bachvaroff, Russell T. Hill, Yantao Li

## ERRATUM

Volume 16, no. 7, e01231-25, 2025, https://doi.org/10.1128/mbio.01231-25. Results section, “Formal proposal of *M. obligatum* gen. nov. sp. nov., *P. aenigmaticus* gen. nov. sp. nov., and associated higher taxonomic ranks," sentence 2: “g__UBA2103” should read “g__1-14-0-10-45-20,” and “g__1-14-0-10-45-20” should read “g__UBA2103.”

Results section, “Description of *M. obligatum,”* sentence 1: “g__1-14-0-10-45-20” should read “g__UBA2103.”

Results section, “Description of *P. aenigmaticus”* sentence 1: “g__UBA2103” should read “g__1-14-0-10-45-20."

