# Supplementary material for: Two novel Patescibacteria: Phycocordibacter aenigmaticus gen. nov. sp. nov. and Minusculum obligatum gen. nov. sp. nov., both associated with microalgae optimized for carbon dioxide sequestration from flue gas
Source: mBio. 2025 Jun 12;16(7):e01231-25. doi: 10.1128/mbio.01231-25 (PMC12239599; doi:10.1128/mbio.01231-25)
Supplement: Supplemental material — Text S1, Figures S1–S12, and Tables S1–S3. [file mbio.01231-25-s0001.pdf]

Supplementary Materials

Two novel Patescibacteria: *Phycocordibacter aenigmaticus* gen. nov. sp. nov. and *Minusculum obligatum* gen. nov. sp. nov., both associated with microalgae optimized for carbon dioxide sequestration from flue gas

Lauren Jonas[a,b], Yi-Ying Lee[a,b], Tsvetan Bachvaroff[a,b], Russell T. Hill[a,b,#] and

Yantao Li[a,b#]

[a] Institute of Marine and Environmental Technology, Baltimore Maryland, USA

[b] University of Maryland Center for Environmental Science, Baltimore Maryland, USA

[#]Corresponding author: hillr@umces.edu

The PDF file includes:

Text S1

Figures S1- S12

Tables S1- S3

Text S1. Description of microalgal growth at 1 L and 500 L scale

Tetradesmus obliquus strain HTB1 was grown with 10% CO_2 and ambient air. A 70 ml seed culture was inoculated in 630 ml BG11 medium, containing 1.76 mM NO_3, 5 mM Ca^{2+}, and 0.01 M $NaHCO_3$. Cultures were maintained at room temperature and exposed to 60 µE m^{-2} s^{-2} light. Cultures were bubbled with either ambient air or air enriched with 10% CO_2 for 14 days. On Day 12, the air enriched with 10% CO_2 was shut off and cultures were bubbled with ambient air until the experiment concluded on Day 14. Microalgal growth was measured through optical density, cell counting with a hemocytometer, dry weight, and ash-free dry weight. Samples were collected on Days 0, 5, 10 (before CO_2 cutoff), and 14 for water chemistry (pH, Ca^{2+}, Mg^{2+}, alkalinity, salinity, and conductivity) and microbiome analysis. *N. oceanica* and *T. obliquus* were also grown in 500 L photobioreactors at Hy-Tek Bio's facility at the Baltimore Back River Wastewater Treatment Plant. The 500 L photobioreactor was illuminated by LEDs from the center of the bioreactor and with three sets of external lights (*ca.* 200 µmol m^{-2} s^{-1} light intensity measured at the mid-point of the bioreactor) (Figure S2). The 500 L culture was sparged with simulated boiler flue gas (5% CO_2) for 27 days. On Day 28 when the algal dry weight plateaued, aeration was switched from 5% CO_2 to ambient air.

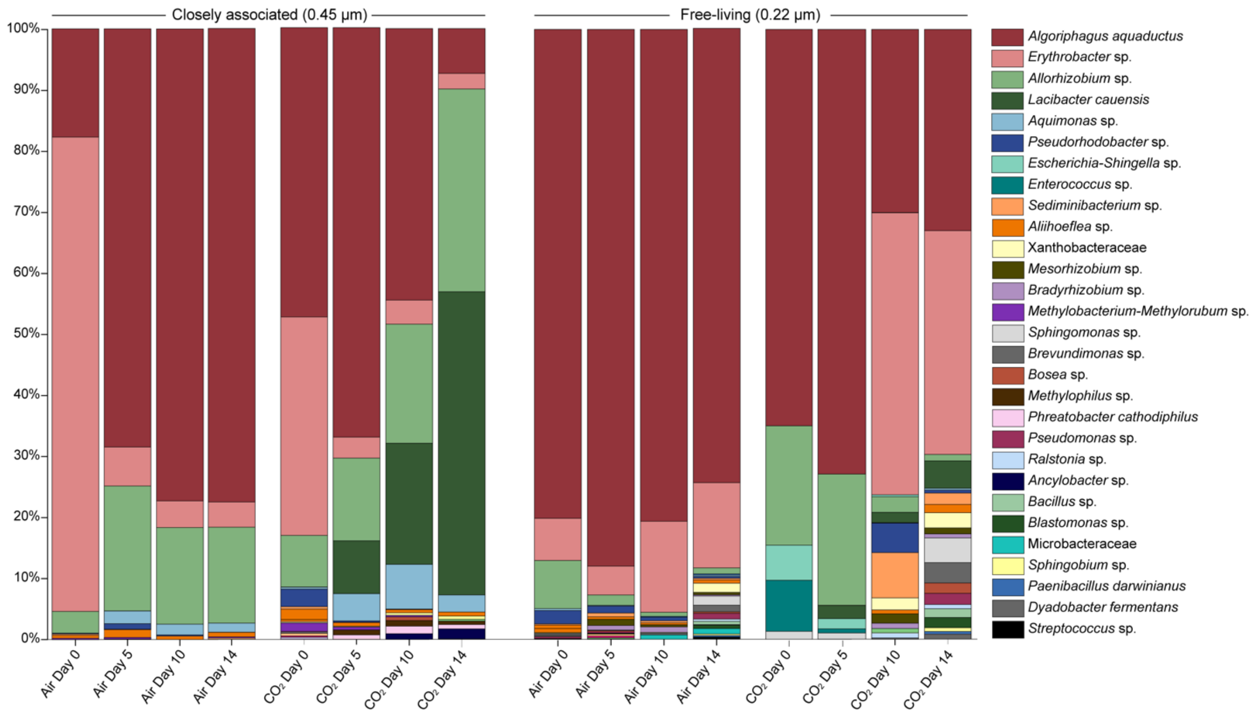

Figure S1. Relative abundance (analysis of the V3/V4 region of the 16S rRNA gene) of closely associated (left 10 panels) and free-living (right 10 panels) bacteria in cultures of *Tetradesmus obliquus* HTB1 grown with ambient air and 10% CO_2. Cultures grown with 10% CO_2 were switched to air on Day 12. 16S rRNA gene sequences from *T. obliquus* chloroplasts have been removed bioinformatically.

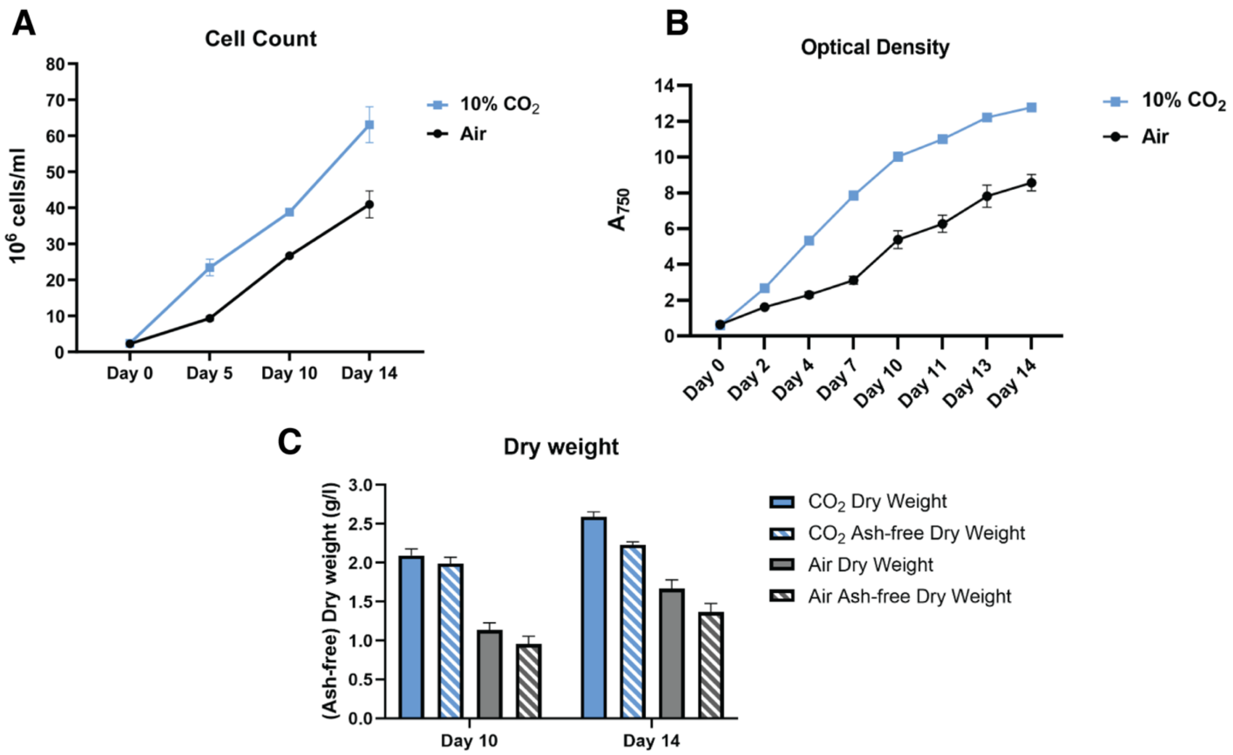

Figure S2. Growth data of *Tetradesmus obliquus* strain HTB1 grown with ambient air (black) and 10% CO_2 (blue) at the 1 L scale. (A) Cell count by hemocytometry, y axis in million cells/milliliter. (B) Optical density by spectrophotometry (Absorbance of 750 nm). (C) Dry weight (solid bars) and Ash-free Dry Weight measurements (striped) in grams/liter.

Table S1. Sequencing statistics of six rounds of Nanopore sequencing. Bolded columns indicate MAGs were recovered from dataset. Mb= million base pairs, Gb= billion base pairs. IMET1 = *Nannochloropsis oceanica* strain IMET. HTB1 = *Tetradesmus obliquus* strain HTB1.

Name	**MAG1-Round1**	**MAG1-Round2**	MAG2	**MAG3**	MAG4	**MAG5**
Sequencing platform	MinION	MinION	GridION	GridION	GridION	GridION
Total bases	**1.43 Gb** **1,427,925,984**	**918 Mb** **918,015,821**	23.3 Mb 232,714,605	**5.45 *Gb*** **5,446,122,139**	195 Mb 195,271,607	**5.14 *Gb*** **5,139,911,566**

Number of reads	**434,869**	**269,260**	71,791	**3.87 Mb** 3,855,281	29,201	**2.46 Mb** 2,461,737
Quality (mean)	**10.7**	**10.9**	14.4	**12.9**	14.5	**12.8**
Quality (median)	**15.8**	**16.8**	16.3	**14.3**	16.5	**14.2**
Length (mean, bp)	**3,283.6**	**3,409.4**	3,241.6	**1,408.6**	6,687.2	**2,087.9**
Length (median, bp)	**2,979**	**3,586**	3,583	**710.0**	5,740	**1,481.0**
N50 Read length (bp)	**3,747**	**3,590**	3,587	**2,910**	10,031	**3,579**
MAGs recovered	**Yes**	**Yes**	No	**Yes**	No	**Yes**
Microalgal culture	**IMET1**	**IMET1**	IMET1	**IMET1**	HTB1	**HTB1**

Table S2. Sequencing statistics before and after filtering for high quality, determining minimum length cut-offs, and removing Nanopore sequencing adapters. Mb= million base pairs, Gb= billion base pairs.

	**MAG Rounds 1 and 2 combined**		**MAG Round 3**		**MAG Round5**	
	**Before processing (raw reads)**	**After removing seqs <100 bp & removing adapters**	**Before processing (raw reads)**	**After removing seqs <100 bp & removing adapters**	**Before processing (raw reads)**	**After removing adapters**
**Total bases**	2.35 Gb 2,345,941,805	2.32 Gb 2,319,637,680	5.45 Gb 5,446,122,139	5.21 Gb 5,306,188,646	5.14 *Gb* 5,139,911,566.0	5.04 *Gb* 5,042,496,571.0
**Number of reads**	704,129	705,774	3.87 million 3,855,281	3.87 million 3,865,115	2.46 million 2,461,737.0	2.46 million 2,462,603.0
**Quality (mean)**	10.8	10.9	12.9	13.7	12.8	13.3
**Quality (median)**	16.2	16.6	14.3	15.3	14.2	15.1
**Length (mean, bp)**	3,331.7	3,286.7	1,408.6	1,372.8	2,087.9	2,047.6
**Length (median, bp)**	3,517	3,487	710	675	1,481.0	1,446
**N50 Read length (bp)**	3,594	3,542	2,910	2,922	3,579	3,529

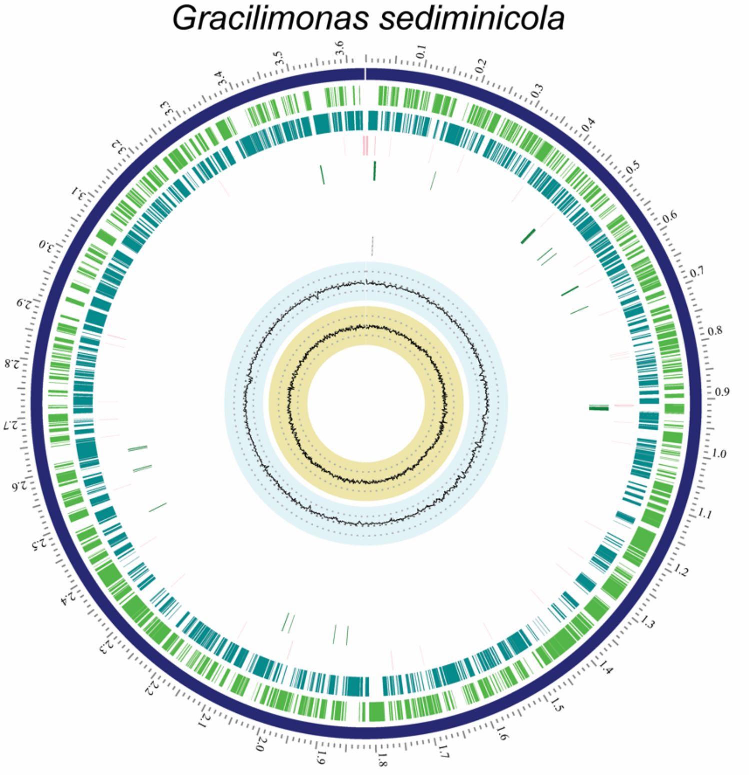

Genome Statistics	
Contigs	1
Genome Length	3629614
GC Content	44.03636
Contig L50	1
Contig N50	3629614
**Annotation Statistics**	
tRNA	42
rRNA	4
CDS	3328
CDS Ratio	0.9169019
Hypothetical CDS	1633
Hypothetical CDS Ratio	0.55288464
**Genome Quality**	
Completeness_Specific (CheckM2)	99.87
Contamination (CheckM2)	0.25

Position Label (Mbp)
Non-CDS Features
Contigs
AMR Genes
CDS- Forward
GC Content
CDS-Reverse
GC Skew

Figure S3. Circular genome view of *Gracilimonas sediminicola*, a MAG recovered from cultures of *Nannochloropsis oceanica* IMET1. Visualization modified from the Bacterial and Viral Bioinformatics Resource Center (BV-BRC). Genome statistics, annotation statistics, and genome completeness and quality are shown in the table to the right.

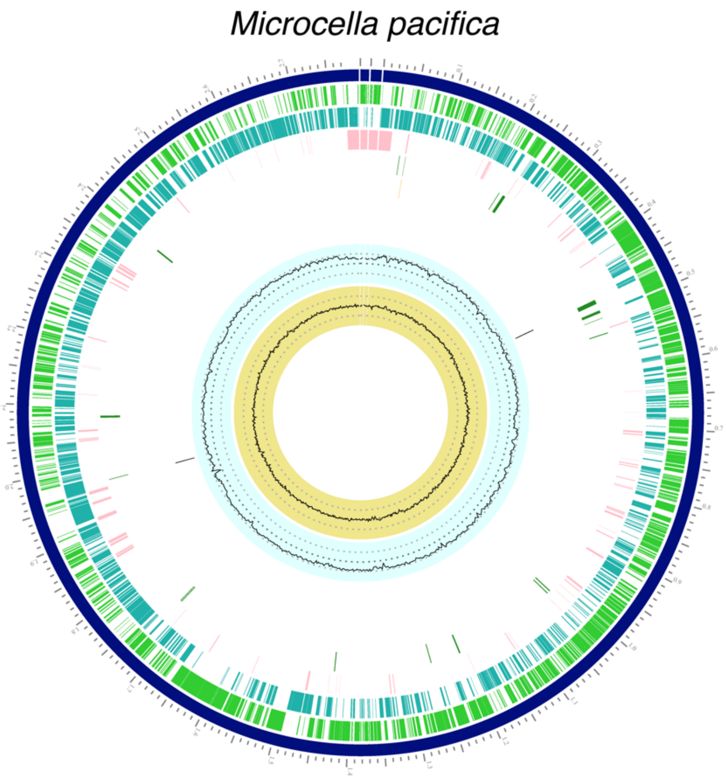

Genome Statistics	
Contigs	3
Genome Length	2818534
GC Content	69.13296
Contig L50	1
Contig N50	2792760
**Annotation Statistics**	
tRNA	45
rRNA	3
CDS	3078
CDS Ratio	1.0920571
Hypothetical CDS	1421
Hypothetical CDS Ratio	0.51786875
**Genome Quality**	
Completeness_Specific (CheckM2)	81.33
Contamination (CheckM2)	1.32

Figure S4. Circular genome view of *Microcella pacifica*, a MAG recovered from cultures of *Nannochloropsis oceanica* IMET1. Visualization modified from the Bacterial and Viral Bioinformatics Resource Center (BV-BRC). Genome statistics, annotation statistics, and genome completeness and quality are shown in the table to the right.

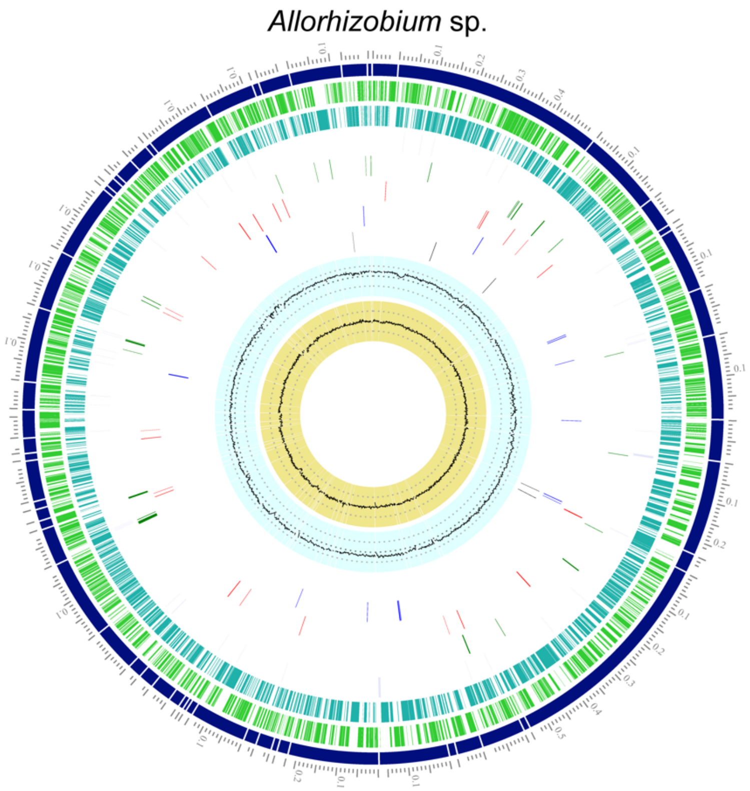

Genome Statistics	
Contigs	57
Genome Length	5109754
GC Content	60.61926
Contig L50	10
Contig N50	168988
**Annotation Statistics**	
tRNA	52
rRNA	6
CDS	5255
CDS Ratio	1.0284252
Hypothetical CDS	1798
Hypothetical CDS Ratio	0.41636536
**Genome Quality**	
Completeness_Specific (CheckM2)	95.74
Contamination (CheckM2)	2.09

Figure S5. Circular genome view of *Allorhizobium sp.*, a MAG recovered from cultures of *Tetradesmus obliquus* HTB1. Visualization modified from the Bacterial and Viral Bioinformatics Resource Center (BV-BRC). Genome statistics, annotation statistics, and genome completeness and quality are shown in the table to the right.

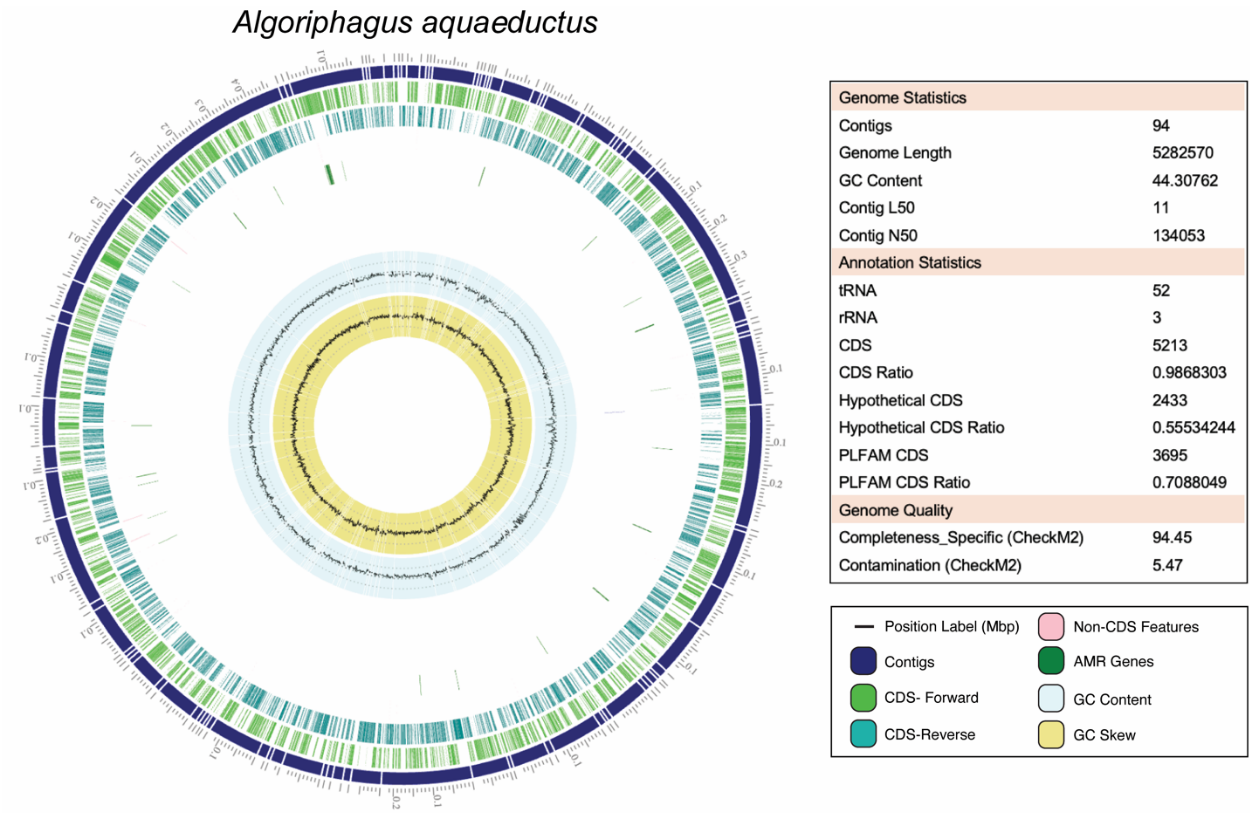

Genome Statistics	
Contigs	94
Genome Length	5282570
GC Content	44.30762
Contig L50	11
Contig N50	134053
**Annotation Statistics**	
tRNA	52
rRNA	3
CDS	5213
CDS Ratio	0.9868303
Hypothetical CDS	2433
Hypothetical CDS Ratio	0.55534244
PLFAM CDS	3695
PLFAM CDS Ratio	0.7088049
**Genome Quality**	
Completeness_Specific (CheckM2)	94.45
Contamination (CheckM2)	5.47

Figure S6. Circular genome view of *Algoriphagus aquaeductus*, a MAG recovered from cultures of *Tetradesmus obliquus* HTB1. Visualization modified from the Bacterial and Viral Bioinformatics Resource Center (BV-BRC). Genome statistics, annotation statistics, and genome completeness and quality are shown in the table to the right.

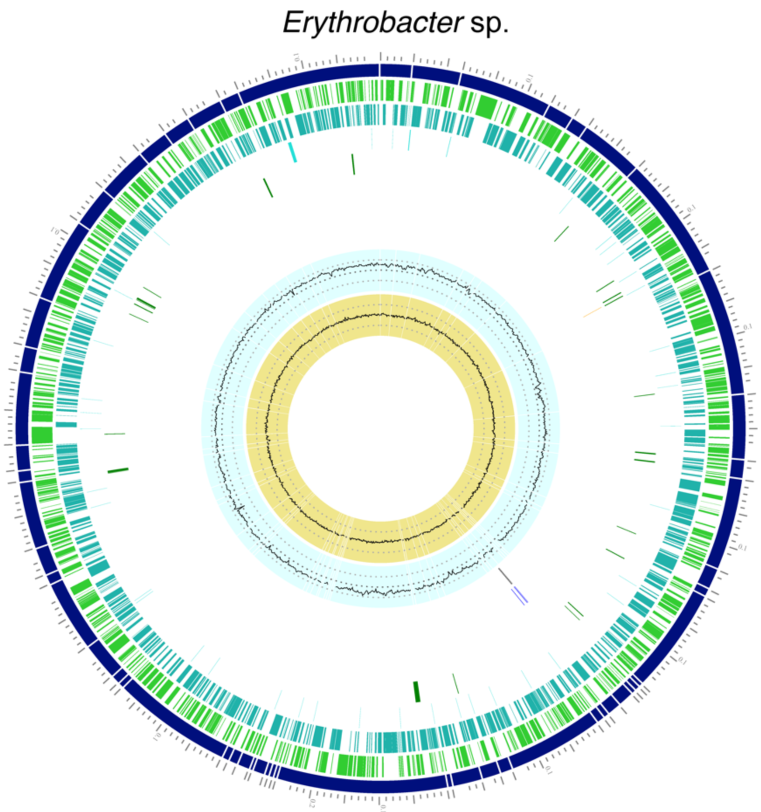

Genome Statistics	
Contigs	56
Genome Length	3211232
GC Content	63.632744
Contig L50	10
Contig N50	121283
**Annotation Statistics**	
tRNA	45
rRNA	3
CDS	3218
CDS Ratio	1.0021076
Hypothetical CDS	1196
Hypothetical CDS Ratio	0.43505284
**Genome Quality**	
Completeness_Specific (CheckM2)	99.31
Contamination (CheckM2)	1.64

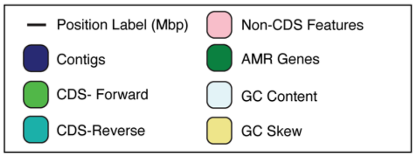

Figure S7. Circular genome view of *Erythrobacter* sp., a MAG recovered from cultures of *Tetradesmus obliquus* HTB1. Visualization modified from the Bacterial and Viral Bioinformatics Resource Center (BV-BRC). Genome statistics, annotation statistics, and genome completeness and quality are shown in the table to the right.

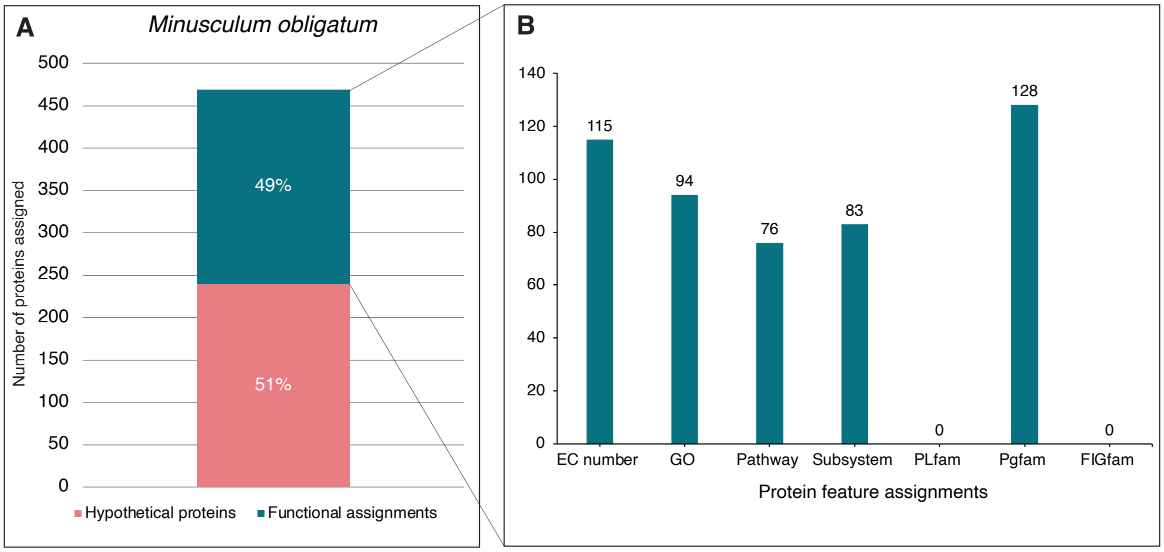

Figure S8. Assignment of protein features in *Minusculum obligatum* by the Pathosystems Resource Integration Center (PATRIC), now provided through Bacterial and Viral Bioinformatics Resource Center (BV-BRC). (A) Percentages of total proteins designated with functional assignments (teal) and hypothetical assignments (pink). (B) Number of protein feature assignments within various databases: EC: Enzyme Commission, GO: Gene Ontology, PLfam: PATRIC genus-specific protein families, PGFam: PATRIC global protein families, FIGfam: custom manually annotated proteins.

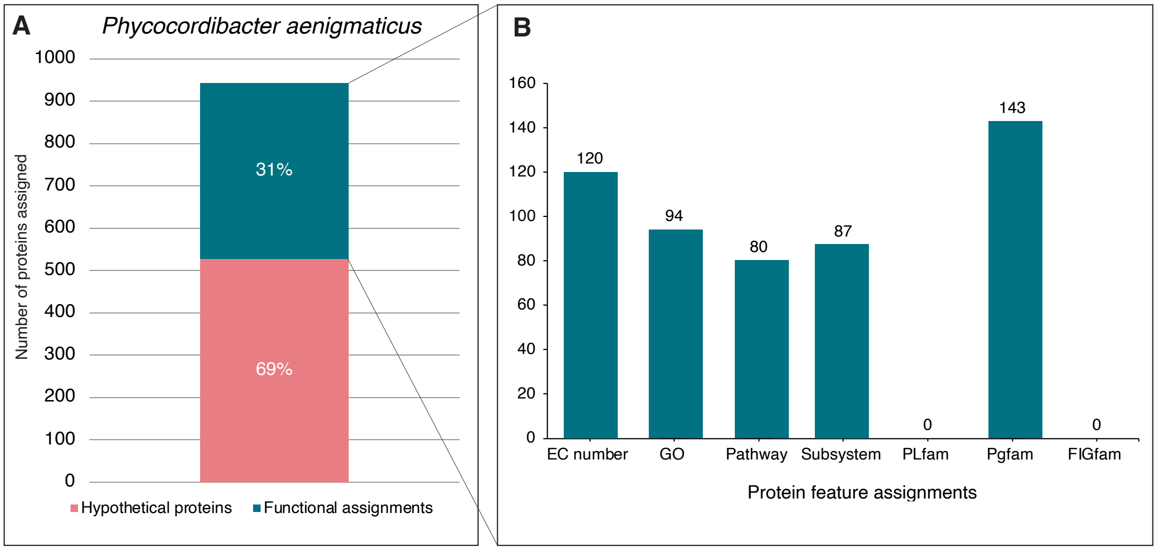

Figure S9. Assignment of protein features in *Phycocordibacter aenigmaticus* by the Pathosystems Resource Integration Center (PATRIC), now provided through Bacterial and Viral Bioinformatics Resource Center (BV-BRC). (A) Percentages of total proteins designated with functional assignments (teal) and hypothetical assignments (pink). (B) Number of protein feature assignments within various databases: EC: Enzyme Commission, GO: Gene Ontology, PLfam: PATRIC genus-specific protein families, PGFam: PATRIC global protein families, FIGfam: custom manually annotated proteins.

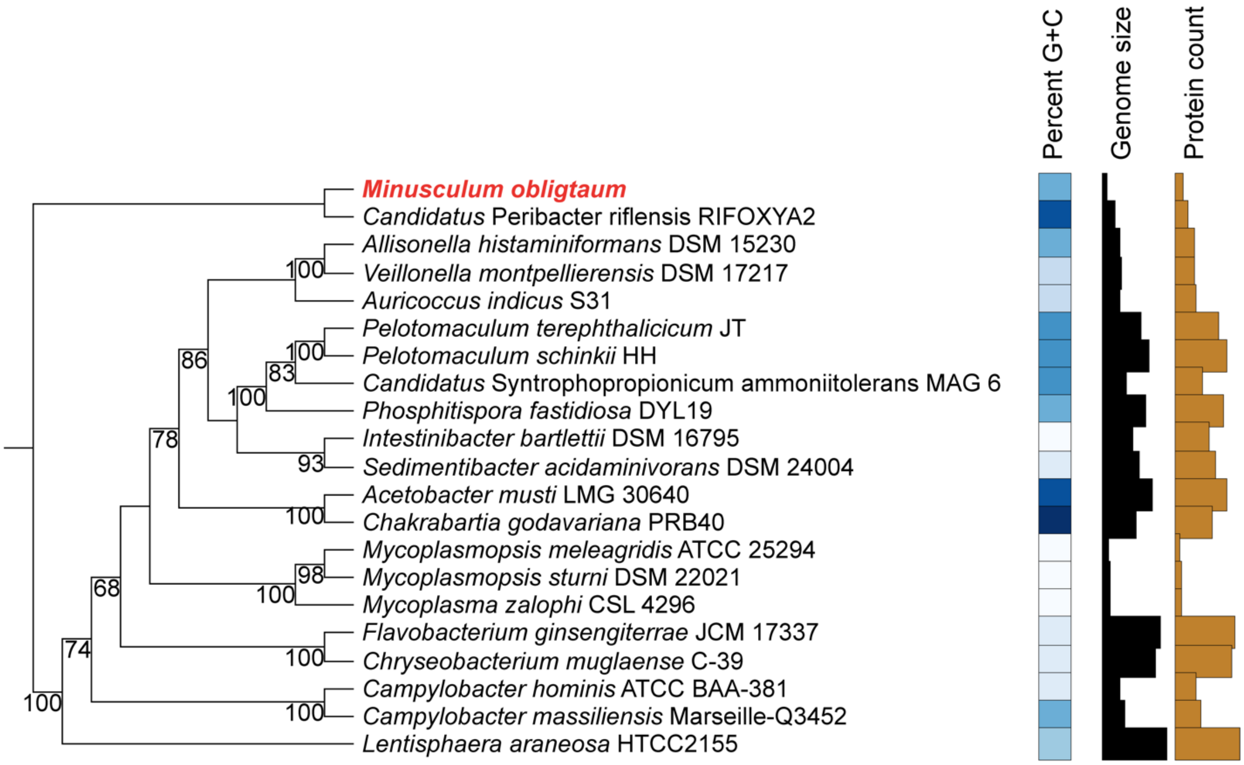

Figure S10. Phylogenetic inference of 16S rRNA genes taken from full genome sequences. *Minusculum obligatum* was compared against all type strain genomes available in the Type Strain Genome Database (TYGS) via the MASH algorithm (1–3). An additional set of ten closely related type strains was determined via the 16S rDNA gene sequences. These were extracted from the user genomes using RNAmmer (4) and each 16S rDNA gene sequence was then alignment to the 22162 type strains available in the TYGS database using BLAST+ (5). Distances were calculated using Genome BLAST Distance Phylogeny approach (GBDP) under the algorithm 'coverage' and distance formula d5 (6). The resulting intergenomic distances were used to infer a balanced minimum evolution tree with branch support via FASTME 2.1.6.1 including SPR postprocessing (7). Branch support was inferred from 100 pseudo-bootstrap replicates each. The trees were rooted at the midpoint (8) and visualized with PhyD3 (9).

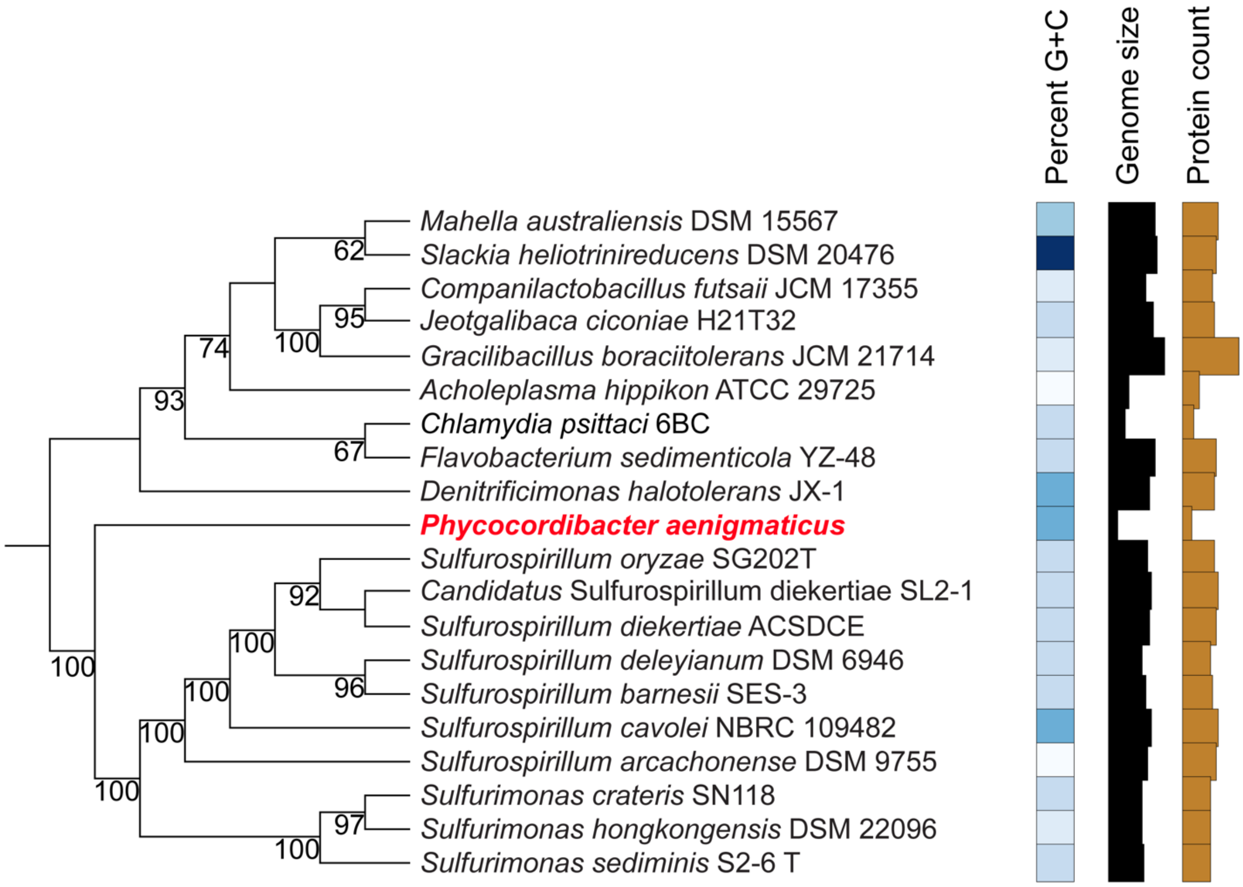

Figure S11. Phylogenetic inference of 16S rRNA genes taken from full genome sequences. *Phycocordibacter aenigmaticus* was compared against all type strain genomes available in the Type Strain Genome Database (TYGS) using the same metrics listed in Figure S10.

Table S3. Raw data output of the symcla lifestyle prediction model. UNI56: percentage of 56 universal bacterial marker genes, results >50% are considered valid. Feature gt01: number of features with a bitscore ≥ 0. Feature ge20: number of features with a bitscore ≥ 20. Feature ge100: number of features with a bitscore ≥ 100. Confidence in symbiont prediction increases with UNI56 completeness and features gt01, ge20, and ge100.

MAG	Completeness UNI56	Features gt01	Features ge20	Features ge100	**Symcla score**	Microalgal host
Minusculum obligatum	91.07	175	107	33	**0.487**	*N. oceanica*
Phycocordibacter aenigmaticus	91.07	189	121	40	**0.548**	*N. oceanica*
Gracilimonas sp.	100	497	335	141	**0.003**	*N. oceanica*
Microcella pacifica	96.43	353	242	99	**0.001**	*N. oceanica*
Erythrobacter sp.	98.21	420	321	134	**-0.001**	*T. obliquus*
Algoriphagus aquaeductus	100	496	344	154	**0.015**	*T. obliquus*
Allorhizobium sp.	87.50	511	388	182	**-0.006**	*T. obliquus*

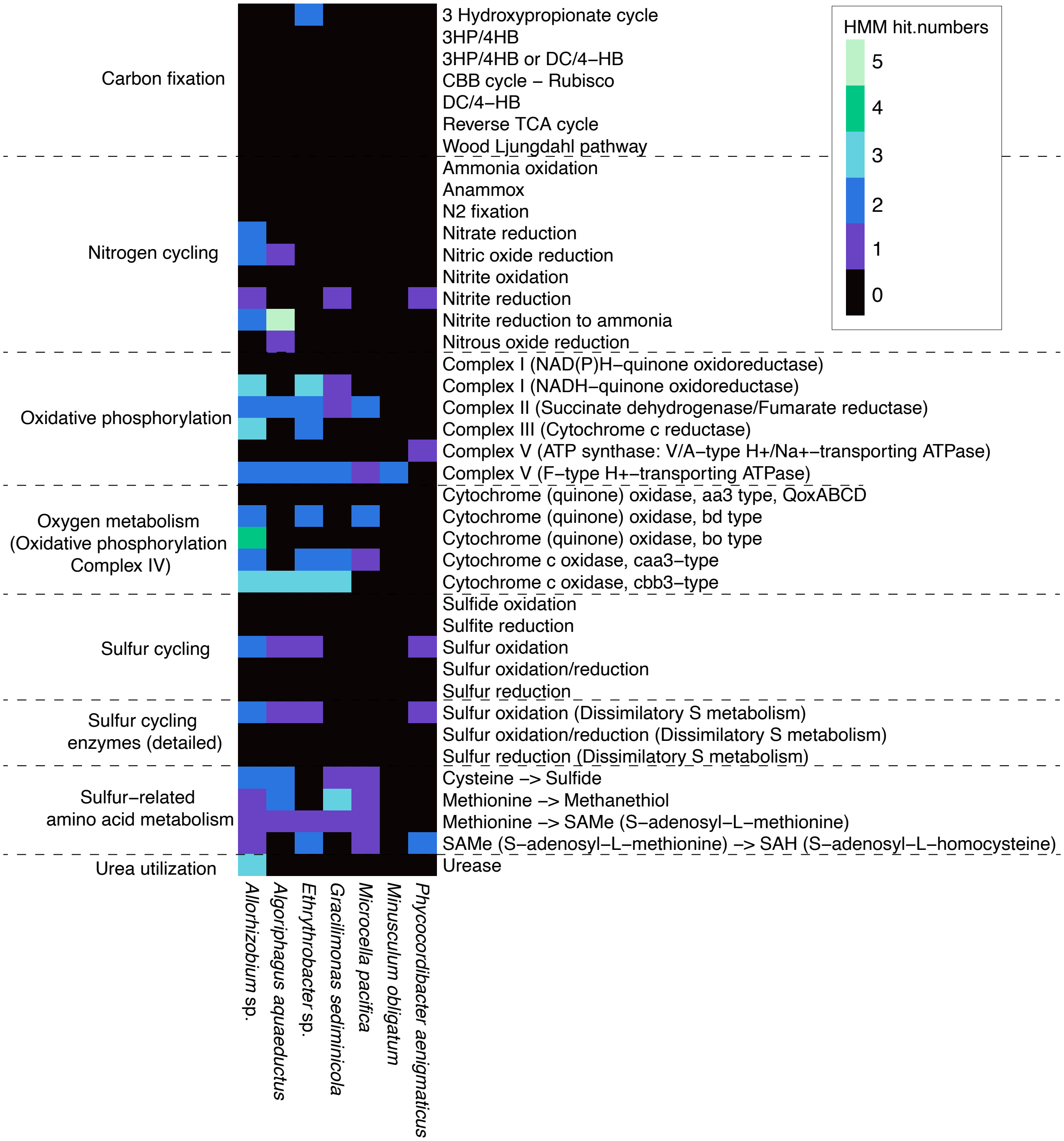

Figure S12. Heatmap displaying metabolic capacity of all seven MAGs predicted with METABOLIC-G (10) querying annotated proteins with Hidden Markov models (HMMs) resulting in custom HMM hit profiles. Individual gene counts within each pathway were summed.

References

1. Meier-Kolthoff JP, Göker M. 2019. TYGS is an automated high-throughput platform for state-of-the-art genome-based taxonomy. Nat Commun 10.
2. Meier-Kolthoff JP, Carbasse JS, Peinado-Olarte RL, Göker M. 2022. TYGS and LPSN: A database tandem for fast and reliable genome-based classification and nomenclature of prokaryotes. Nucleic Acids Res 50.
3. Ondov BD, Treangen TJ, Melsted P, Mallonee AB, Bergman NH, Koren S, Phillippy AM. 2016. Mash: Fast genome and metagenome distance estimation using MinHash. Genome Biol 17.
4. Lagesen K, Hallin P, Rødland EA, Stærfeldt HH, Rognes T, Ussery DW. 2007. RNAmmer: Consistent and rapid annotation of ribosomal RNA genes. Nucleic Acids Res 35.
5. Camacho C, Coulouris G, Avagyan V, Ma N, Papadopoulos J, Bealer K, Madden TL. 2009. BLAST+: Architecture and applications. BMC Bioinformatics 10.
6. Meier-Kolthoff JP, Auch AF, Klenk HP, Göker M. 2013. Genome sequence-based species delimitation with confidence intervals and improved distance functions. BMC Bioinformatics 14.
7. Lefort V, Desper R, Gascuel O. 2015. FastME 2.0: A comprehensive, accurate, and fast distance-based phylogeny inference program. Mol Biol Evol 32.
8. Farris JS. 1972. Estimating phylogenetic trees from distance matrices. Am Nat 106.
9. Kreft L, Botzki A, Coppens F, Vandepoele K, Van Bel M. 2017. PhyD3: A phylogenetic tree viewer with extended phyloXML support for functional genomics data visualization. Bioinformatics 33.

10. Zhou Z, Tran PQ, Breister AM, Liu Y, Kieft K, Cowley ES, Karaoz U, Anantharaman K. 2022. METABOLIC: high-throughput profiling of microbial genomes for functional traits, metabolism, biogeochemistry, and community-scale functional networks. Microbiome 10.
